# ADARs regulate cuticle collagen expression and promote survival to pathogen infection

**DOI:** 10.1186/s12915-024-01840-1

**Published:** 2024-02-16

**Authors:** Alfa Dhakal, Chinnu Salim, Mary Skelly, Yarden Amichan, Ayelet T. Lamm, Heather A. Hundley

**Affiliations:** 1 Cell, Molecular and Cancer Biology Graduate Program, Indiana University School of Medicine-Bloomington, Bloomington, IN 47405 USA; 2 Department of Biology, Indiana University, Bloomington, IN 47405 USA; 3 Faculty of Biology, Technion Institute of Technology, Haifa, Israel

**Keywords:** Innate immunity, RNA editing, Double-stranded RNA (dsRNA), *C. elegans*, *P. aeruginosa*, RNA modification, Post-transcriptional regulation, RNA-binding protein

## Abstract

**Background:**

In all organisms, the innate immune system defends against pathogens through basal expression of molecules that provide critical barriers to invasion and inducible expression of effectors that combat infection. The adenosine deaminase that act on RNA (ADAR) family of RNA-binding proteins has been reported to influence innate immunity in metazoans. However, studies on the susceptibility of ADAR mutant animals to infection are largely lacking.

**Results:**

Here, by analyzing *adr-1* and *adr-2* null mutants in well-established slow-killing assays, we find that both *Caenorhabditis elegans* ADARs are important for organismal survival to gram-negative and gram-positive bacteria, all of which are pathogenic to humans. Furthermore, our high-throughput sequencing and genetic analysis reveal that ADR-1 and ADR-2 function in the same pathway to regulate collagen expression. Consistent with this finding, our scanning electron microscopy studies indicate *adr-1*;*adr-2* mutant animals also have altered cuticle morphology prior to pathogen exposure.

**Conclusions:**

Our data uncover a critical role of the *C. elegans* ADAR family of RNA-binding proteins in promoting cuticular collagen expression, which represents a new post-transcriptional regulatory node that influences the extracellular matrix. In addition, we provide the first evidence that ADAR mutant animals have altered susceptibility to infection with several opportunistic human pathogens, suggesting a broader role of ADARs in altering physical barriers to infection to influence innate immunity.

**Supplementary Information:**

The online version contains supplementary material available at 10.1186/s12915-024-01840-1.

## Background

Pathogen infection is a major environmental threat that results in agricultural devastation and economic loss and serves as a major cause of human mortality/morbidity. To counter these attacks, plants and animals employ both physical barriers and physiological responses to resist and kill invading pathogens [1]. The most well studied innate immune responses are the evolutionary conserved signaling pathways, wherein the pathogenic “signal” is recognized by the host and triggers gene expression changes that produce cellular effectors capable of promoting organismal survival [2, 3]. Roles for RNA-binding proteins (RBPs) in modulating pathogenic signal recognition have been examined [4, 5], particularly for viral infection as RNA can be the carrier of viral genomic information.

Members of the adenosine deaminase that act on RNA (ADAR) family of RBPs have well-established roles during viral infection [6]. The initial focus on ADARs and virus infection was in large part because double-stranded RNA (dsRNA) is the substrate of ADARs, and dsRNA was initially thought to be unique to the genomes of some viruses and/or formed during the viral lifecycle. However, through studies of ADAR cellular targets, it has become clear that metazoan transcriptomes are ripe with dsRNA regions [7, 8]. ADARs bind dsRNA and can change the dsRNA sequence and structure via catalyzing deamination of adenosine (A) to inosine (I), a process commonly referred to as A-to-I RNA editing [9]. Editing of cellular dsRNAs is essential in mammals for both diversification of the nervous system proteome and to prevent the aberrant interaction of cellular transcripts with dsRNA sensors of the innate immune pathway [10]. This later function was uncovered after ADAR mutations were identified in patients suffering from autoimmune disorders [11], and additional studies demonstrated that loss of dsRNA sensors rescues lethality of ADAR mutations in mice [12–14]. Furthermore, as ADARs are conserved in metazoans, studies from several model organisms have explored these relationships and provide data that link ADAR loss with changes in immune gene expression [15–17]. However, studies on the susceptibility of ADAR mutant animals to infection are largely lacking.

In this work, we sought to determine the effect of loss of *Caenorhabditis elegans* ADARs on susceptibility to pathogen infection. The *C. elegans* genome encodes two ADAR family members, ADR-1 and ADR-2 [18]. While both genes contain the canonical ADAR domain structure, ADR-2 is the sole enzyme providing A-to-I editing activity in *C. elegans* [19]. However, loss of *adr-1* impacts both RNA editing and expression of edited genes during development [20, 21]. Recent studies have indicated that combined loss of both *adrs* and small RNA processing factors led to altered upregulation of antiviral genes and developmental defects, including vulva morphology defects and frequent bursting [22, 23]. However, neither study addressed sensitivity or resistance of the mutant animals to infection. Furthermore, the upregulated genes in the animals lacking *adrs* and small RNA processing factors overlapped not only with those regulated by viral infection, but also infection with intracellular pathogens and other general stress responses [23]. In fact, data from many recent studies, particularly in the model organism *C. elegans*, has indicated that innate immune responses are intertwined with different homeostatic mechanisms, such as the unfolded protein response as well as germline integrity [24].

To directly address the physiological role of ADARs in innate immunity, survival of *adr* mutant animals to pathogenic infection was assessed using well-established assays with several bacterial species, all of which are pathogenic to humans. Our data demonstrates that animals lacking ADARs exhibit enhanced susceptibility to pathogenic infection. Furthermore, our gene regulatory analysis and scanning electron microscopy studies indicate that *adr* mutant animals have decreased collagen expression and altered cuticle morphology. As employment of physical barriers is also critical to resisting invading pathogens, these data suggest that the role of ADARs in innate immunity may not be limited to altering dsRNA structures to prevent aberrant activation of immune response.

## Results

### Loss of *adr-1* or *adr-2* increases sensitivity of *C. elegans* to *Pseudomonas aeruginosa*

**Fig. 1:**
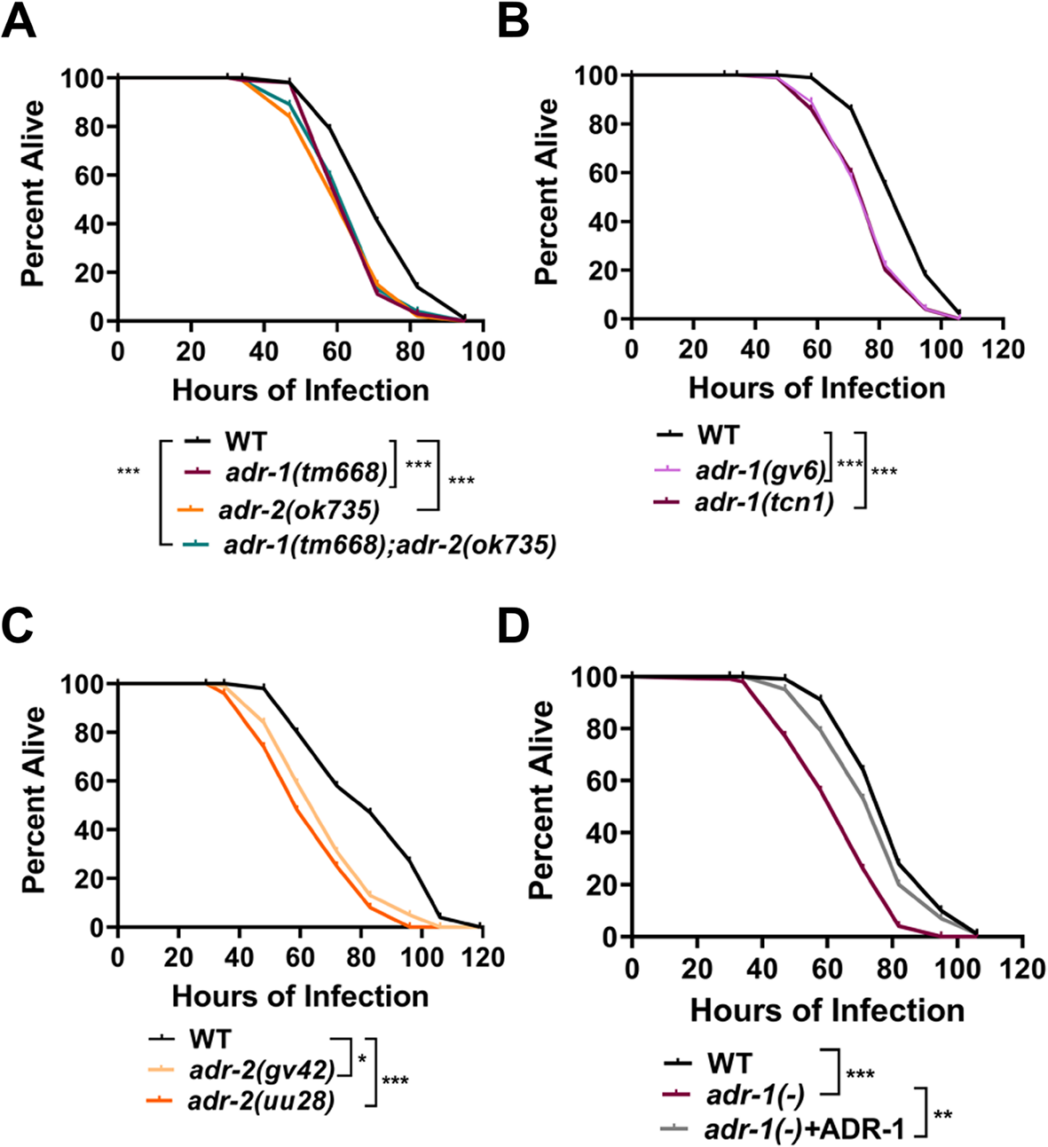
ADAR mutant worms are susceptible to *Pseudomonas* infection. **A**–**D** Representative survival curve (of three biological replicates) for the indicated animals subjected to the slow-killing assay and scored for survival in response to *P. aeruginosa* strain PA14. Statistical significance determined using OASIS. *p* < 0.001 (***), *p* < 0.001 (**) and *p* < 0.05 (*). Survival assay replicates are provided in Additional file 1: Fig: S1A-F

To determine whether *C. elegans* ADR-1 and ADR-2 influence survival to pathogen infection, survival was assessed using a well-established assay with the gram-negative bacterium, *Pseudomonas aeruginosa. P. aeruginosa* is an opportunistic pathogen causing both acute and chronic infection in patients with cystic fibrosis, burn wounds and other diseases requiring ventilation, such as COVID-19 [25]. Similar to humans, *P. aeruginosa* can infect and kill *C. elegans* [26]. Using a standard slow-killing assay, survival of animals lacking *adr-1*, *adr-2* or both genes was assessed on plates containing a small lawn of the PA14 clinical isolate of *P. aeruginosa* (Fig. 1A). As expected, wildtype animals exposed to *P. aeruginosa* die over the course of several days (Fig. 1A). Animals lacking *adr-1* or *adr-2* showed a reproducible and significant sensitivity to killing by *P. aeruginosa* (Fig. 1A, Additional file 1: Fig. S1A, B). Furthermore, animals lacking both *adr-1* and *adr-2* had a similar survival as the animals lacking the individual *adrs* (Fig. 1A, Additional file 1: Fig. S1A, B), suggesting the two *adrs* are functioning together to promote organismal resistance to *P. aeruginosa* infection.

It is important to note that previous studies have indicated alterations in lifespan of *adr* mutant animals begin to occur around 10 days after L4 worms were grown in the presence of standard nematode bacterial food (*E. coli* OP50) and reach approximately 50% survival around 20–30 days [20, 27]. Although the lifespan phenotypes reported differed for the individual *adr-1(-)* and *adr-2(-)* animals, with *adr-2(-)* being long-lived and *adr-1(-)* animals being short-lived [20], to test whether the reported growth defects impact general survival of *adr* mutant animals during the timing and growth of the pathogenic survival assays, survival was analyzed using the standard slow-killing assay method but with OP50 as the food source. Under these conditions, survival of the *adr-1(-)*, *adr-2(-)*, and *adr-1(-);adr-2(-)* mutant animals was similar to wildtype animals (Additional file 2: Fig. S2). However, consistent with our initial results, *adr-1(-);adr-2(-)* mutant animals monitored in the same biological replicates for growth in the presence of *P. aeruginosa* showed a significant decrease in survival compared to wildtype animals (Additional file 2: Fig. S2). Together, these results suggest that both ADR-1 and ADR-2 play a role in regulating animal survival to pathogenic bacteria. However, it is also possible that *adr* mutant animals exhibit enhanced susceptibility due to an increased general sensitivity to acute stressors. To begin to test this possibility, an acute thermal stress assay [28] was performed. Briefly, wildtype and *adr* mutant animals were synchronized and grown to the L4 stage in the same manner as for the slow-killing assay. However, instead of exposing to *P. aeruginosa*, animals were exposed to 35 °C for 6 h and survival was assessed after 14 h of recovery at 20 °C (Additional file 3: Fig. S3). While approximately 20% of wildtype animals die after the acute heat stress, only 10% of *adr* mutant animals die after the same 35 °C stress. These data indicate that the individual *adr-1(-)* and *adr-2(-)* animals as well as the *adr-1(-)*;*adr-2(-)* animals are not sensitive to acute heat stress and support our findings that both ADARs are important for organismal survival to pathogenic bacteria.

To rigorously test the impact of loss of *adr-1* and *adr-2* for survival to *P. aeruginosa*, multiple, different deletion alleles were examined. For *adr-1*, survival was assessed for the established *adr-1(gv6)* animals [19] and a newly created CRISPR allele of *adr-1(tcn1)*, which has a complete deletion of the *adr-1* coding sequence (Fig. 1B, Additional file 1: Fig. S1C, D). For *adr-2*, survival was assessed for the established *adr-2(gv42)* [19] and *adr-2(uu28)* [22] animals (Fig. 1C, Additional file 1: Fig. S1E, F). Importantly, these four additional mutant strains all resulted in reproducible and significant sensitivity to killing by *P. aeruginosa* (Fig. 1B, C. Additional file 1: Fig. S1C-F). As a secondary approach, the pathogen susceptibility of an *adr-1(-)* strain carrying a transgene expressing *adr-1* under the control of the *adr-1* promoter was examined (Fig. 1D, Additional file 1: Fig. S1G, H). Re-introduction of *adr-1* into *adr-1(tm668)* animals significantly improved survival to *P. aeruginosa* (Fig. 1D, Additional file 1: Fig. S1G, H). Transgenic rescue lines for *adr-2(-)* animals have been unsuccessful to date (unpublished results), likely due to the presence of *adr-2* as the second gene in a six-gene operon [29]. However, consistent with the requirement for each *adr* in survival to *P. aeruginosa*, the presence of the *adr-1* transgene described above could not rescue the defect of *adr-1(-);adr-2(-)* animals (see Additional file 4: Fig. S4). Together, these data indicate that both ADR-1 and ADR-2 are important and function in the same pathway to promote *C. elegans* survival to *P. aeruginosa* infection.

### *adr* mutant animals exhibit normal avoidance and feeding behavior to *P. aeruginosa*

**Fig. 2:**
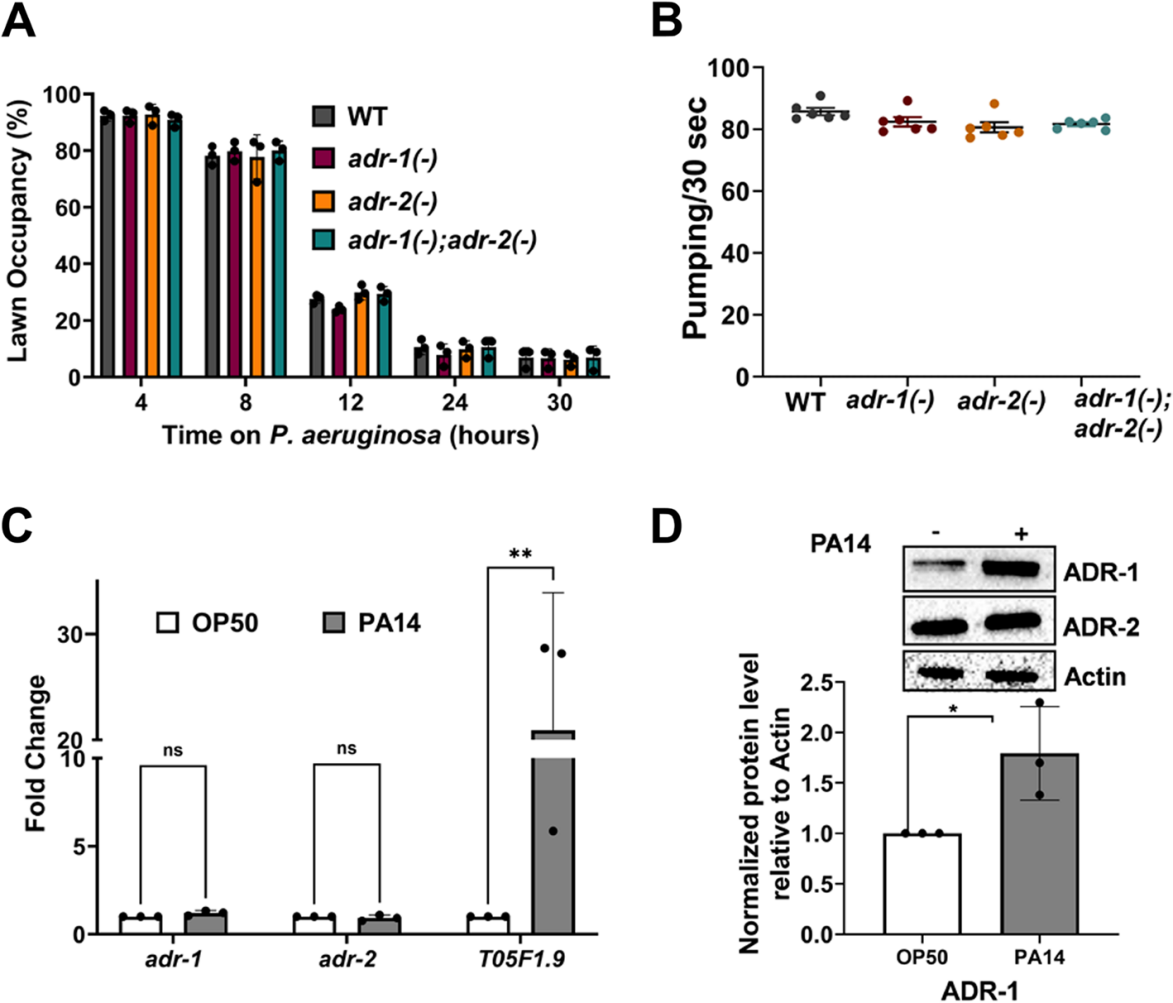
*Adr* mutant animals exhibit normal avoidance and feeding behavior to *P. aeruginosa.* **A** Lawn occupancy percentage was calculated by counting the number of worms of the indicated strains in the lawn and outside the lawn, which was summed and then divided by the total number of worms. Each data point represents the average of three technical replicates performed at the indicated time and all experiments were performed in three biological replicates. Error bars represent the standard error of the mean (SEM). Statistical significance determined using two-way ANOVA Tukey’s multiple comparisons test. *p* value of each of the mutants relative to WT was not significant (*p* > 0.05) for any of the timepoints. **B** Each dot in the graph represents the average pumping rate for three technical replicates obtained in two independent experiments. Error bars represent SEM. Statistical significance determined using unpaired Mann Whitney test, with no significant differences observed between wildtype and the *adr* mutant animals (*p* > 0.05). **C** qRT-PCR quantification of the level of the indicated genes relative to *gpd-3* and normalized to the ratios obtained for OP50. The mean of three biological replicates was plotted. Error bars represent SEM. Statistical significance determined using a two-way ANOVA Sidak’s multiple comparisons test. *p ≤ 0.05, **p ≤ 0.005, ns indicates no significant difference (*p* > 0.05). **D** Equivalent amounts of lysate from animals with V5 and 3xFLAG epitope tags on ADR-1 and ADR-2, respectively, exposed to *P. aeruginosa* (PA14) ( +) or the control *E. coli* (OP50) ( −) for 7 h were subjected to SDS-PAGE and immunoblotting with V5 (ADR-1), FLAG (ADR-2), and Actin antibodies. Raw blot images of all three replicates are provided in Additional file 6: Fig. S6

Organismal survival to pathogen infection involves both critical gene regulatory programs as well as behavioral responses, such as movement away from the pathogen [30–32]. As *adr* mutant animals have altered chemotactic behavior [19], it is possible that the decreased survival is an indirect effect caused by the inability to sense *P. aeruginosa*. To directly test this possibility, occupancy of wildtype and *adr* mutant animals within the small lawn of *P. aeruginosa* was monitored at five different timepoints during the first 30 h of exposure. Importantly, there is no significant difference in survival of wildtype and *adr* mutant animals during these first hours of exposure (Fig. 1A). Consistent with previous studies [33], in the first 8 h of exposure, most wildtype animals do not have a strong preference to avoid *P. aeruginosa*; however, between 12 and 30 h after exposure, wildtype animals spend more time off the bacterial lawn than within the lawn (Fig. 2A). There was no significant difference between the occupancy of wildtype and *adr* mutant animals at any point during the assay (Fig. 2A). These data suggest that decreased survival of *adr* mutant animals exposed to *P. aeruginosa* is not caused by an inability to avoid pathogen.

It is also possible that *adr* mutant animals are more susceptible due to increased *P. aeruginosa* intake. To monitor intake, pharyngeal pumping was measured for animals after 24 h of exposure to *P. aeruginosa*. Pumping rates observed for wildtype animals were similar to those previously reported [34], and *adr* mutant animals did not have significantly different pharyngeal pumping rates when compared to wildtype animals (Fig. 2B). This suggests a similar level of pathogen intake in all the strains and that the enhanced susceptibility of the *adr* mutant worms is likely not due to more intake of *P. aeruginosa*.

As *adr* mutant animals did not appear to have defects in pathogen avoidance or intake, it is possible that the susceptibility arises because, in wildtype animals, ADR-1 and ADR-2 are critical effectors that increase expression upon pathogen exposure, a feature lost in *adr* mutant animals. To examine this possibility, ADAR protein and mRNA levels were analyzed in response to *P. aeruginosa* infection. As activation of immune response genes occurs within 4 to 8 h after exposure to *P. aeruginosa* [35, 36], wildtype animals were subjected to a 7-h exposure followed by RNA and protein isolation. To facilitate detection of protein levels, wildtype animals were CRISPR modified to express a V5 epitope at the N-terminus of ADR-1 and three copies of the FLAG epitope at the N-terminus of ADR-2. The epitope tags did not affect known behavioral consequences caused by lack of *adr* function or RNA editing (Additional file 5: Fig. S5A, B). To confirm activation of the immune response, expression of *T05F1.9*, a gene previously shown to be upregulated by *P. aeruginosa* exposure [36] was analyzed by quantitative real-time PCR (qRT-PCR) in three biological replicates of RNA isolated from animals exposed to *P. aeruginosa* and compared to RNA isolated from the same animals grown on plates with the standard *C. elegans* bacterial food source*, E. coli* strain OP50. In contrast to *T05F1.9*, both *adr-1* and *adr-2* mRNA levels did not change upon exposure to *P. aeruginosa* (Fig. 2C). Consistent with the mRNA levels, ADR-2 expression did not change upon *P. aeruginosa* exposure (Fig. 2D). In contrast, ADR-1 protein expression significantly increased upon *P. aeruginosa* exposure (Fig. 2D, Additional file 6: Fig. S6), suggesting that ADR-1 expression may be post-transcriptionally controlled when animals encounter a bacterial pathogen.

### ADR-1 RNA binding is required for survival to *P. aeruginosa* infection

**Fig. 3:**
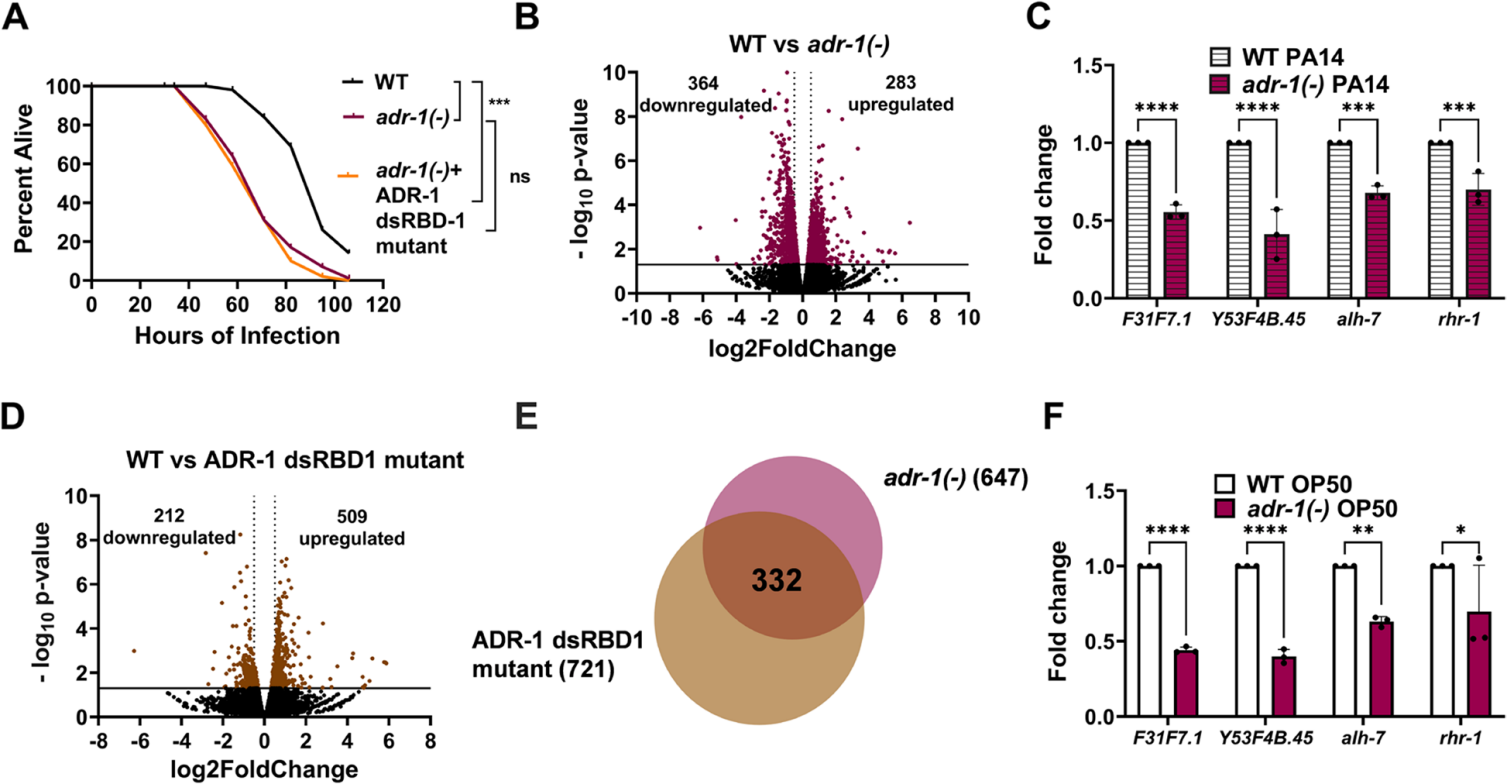
ADR-1 RNA binding is required for survival to *P. aeruginosa.* **A** Representative survival curve (of three biological replicates) for the indicated animals subjected to the slow-killing assay and scored for survival in response to *P. aeruginosa* strain PA14. Statistical significance determined using OASIS, where *p* < 0.001 (***), and ns indicates *p* > 0.05. Survival assay replicates are provided in Additional file 7: Fig. S7. Dots represent individual genes that are down- or upregulated or not significantly differentially expressed (black) in RNA-seq data from WT animals compared to *adr-1(-)* (maroon) (**B**) or ADR-1 dsRBD1 mutant animals (brown). **D** Average log_2_ fold change (*x*-axis) is plotted against the negative log_10_ *p*-values (*y*-axis). Genes considered significantly differentially expressed exhibited log_2_ fold change of |0.5| (light gray dotted vertical lines) and* p* < 0.05 [ log_10_ *p-*value of 1.3, solid black horizontal line]. **C**,** F** qRT-PCR quantification of the level of the indicated genes relative to *gpd-3* and normalized to the ratios obtained for WT PA14 and WT OP50 in (**C** and **F**), respectively. The mean of three biological replicates was plotted. Error bars represent SEM. Statistical significance was determined using a two-way ANOVA Sidak’s multiple comparisons test. *p ≤ 0.05, **p ≤ 0.005, ***p ≤ 0.0005, ****p ≤ 0.0001, ns indicates no significant difference (*p* > 0.05). **E** Overlap of genes misregulated in (**B** and **D**)

The upregulation of ADR-1 after *P. aeruginosa* exposure suggests ADR-1 may play a role in response to infection. While lacking deaminase activity [19], ADR-1 does possess double-stranded RNA (dsRNA) binding activity [37]. The impacts of loss of ADR-1 RNA binding on gene expression are not known. However, RNA binding by ADR-1 is known to both positively and negatively regulate ADR-2-mediated RNA editing, depending on the tissue, developmental timing and specific transcript [21, 38]. To investigate the role of ADR-1 RNA binding in survival of animals exposed to *P. aeruginosa*, the survival assay was performed with *adr-1(-)* animals containing an extrachromosomal array expressing an ADR-1 dsRBD1 mutant under the control of the *adr-1* promoter. The ADR-1 dsRBD1 mutant has EAxxA (E = glutamic acid, A = alanine and x = any amino acid) present in place of the conserved KKxxK (K = lysine) motif and previous studies have indicated that this ADR-1 dsRBD1 mutant lacks the ability to bind known ADR-1 mRNA targets in vivo [37]. Consistent with our earlier results (Fig. 1A), survival of *adr-1(-)* animals exposed to *P. aeruginosa* infection was significantly shorter than wildtype animals (Fig. 3A). However, in contrast to the ability of transgenic wildtype *adr-1* to restore survival to *adr-1(-)* animals (Fig. 1D), survival of the ADR-1 dsRBD1 mutant animals was not significantly different from *adr-1(-)* animals (Fig. 3A, Additional file 7: Fig. S7). These data suggest that ADR-1 binding to mRNA is important for survival to *P. aeruginosa* infection.

To further investigate the role of ADR-1 in survival to *P. aeruginosa* infection, we sought to identify transcripts regulated by ADR-1 mRNA binding in response to *P. aeruginosa* infection. To this end, high-throughput sequencing was performed on polyadenylated RNA isolated from wildtype, *adr-1(-)* and the ADR-1 dsRBD1 mutant animals after 7 h of exposure to *P. aeruginosa*. When compared to wildtype animals, differential gene expression analysis of two biological replicates revealed 647 significantly differentially expressed genes (*p* < 0.05, log_2_fold change >|0.5|) in the *adr-1(-)* RNA-sequencing (RNA-seq) data (Fig. 3B, Additional file 8: Table S8). Among the differentially expressed genes, there were 283 up- and 364 downregulated genes (Fig. 3B). To independently validate the RNA-seq findings, four genes identified as differentially expressed were randomly chosen and analyzed by qRT-PCR in three independent biological replicates. Consistent with the RNA-seq data, all four genes (*F31F7.1*, *Y53F4B.45*, *alh-7* and *rhr-1*) were significantly downregulated in RNA isolated from *adr-1(-)* animals exposed to *P. aeruginosa* when compared to RNA isolated from wildtype animals exposed to *P. aeruginosa* (Fig. 3C).

To determine how many differentially expressed genes are directly regulated by ADR-1 binding, the wildtype and ADR-1 dsRBD1 mutant RNA-seq datasets were compared and overlapped with the genes misregulated in the absence of *adr-1*. Differential gene expression analysis revealed 721 significantly differentially expressed (*p* < 0.05, log_2_fold change >|0.5|) genes between the ADR-1 dsRBD1 mutant RNA-seq data and the wildtype RNA-seq data (Fig. 3D, Additional file 9: Table S9). Importantly, nearly half of these misregulated genes (332/721) are observed in our datasets of differentially regulated genes from *adr-1(-)* animals (Fig. 3E, Additional file 9: Table S9), suggesting that loss of ADR-1 binding to mRNA plays a major role in ADR-1-mediated control of gene expression. While human ADARs have been shown to have editing-independent, RNA binding-dependent gene regulatory functions on a handful of genes [39], our high-throughput sequencing analysis provides the first direct evidence that RNA binding by an ADAR family member significantly contributes to altered mRNA expression.

To assess the contribution of the newly identified genes controlled by ADR-1 RNA binding to the pathogen susceptibility phenotype, these targets were compared to known *C. elegans* pathogen response genes. Surprisingly, very few (21/332) of the ADR-1 regulated genes were also previously shown to be upregulated in wildtype worms exposed to *P. aeruginosa* [36] (Dataset accession number: GSE5793) (Additional file 9: Table S9). A similar trend of minimal overlap (36/648) was also observed with genes only misregulated in *adr-1(-)* animals (Additional file 8: Table S8). Furthermore and consistent with a lack of global immune gene upregulation, there was also minimal overlap with ADR-1 and ADR-1 dsRBD mutant co-regulated genes regulated in response to *Staphylococcus aureus* [40] (Dataset accession number: GSE2405) (30/332, Additional file 9: Table S9) or *Enterococcus faecalis* [41] (Dataset accession number: GSE95636) (13/332, Additional file 9: Table S9).

To perform a quantitative and unbiased search of enriched gene sets of the ADR-1/ADR-1 dsRBD mutant co-regulated genes, the *C. elegans*-specific software, WormCat [42] was employed. This gene set enrichment analysis (GSEA) of the 332 transcripts regulated by loss of *adr-1* and loss of ADR-1 RNA binding did not detect significant enrichment of categories related to the innate immune response or other defense functions (Additional file 10: Fig. S10A). A second complementary ontology analysis was performed with the FuncAssociate software [43], previously employed in studies of pathogenic infection in *C. elegans* [40]. Consistent with the WormCat analysis, the FuncAssociate analysis of the 332 transcripts regulated by loss of *adr-1* and loss of ADR-1 RNA binding did not identify enrichment for immune regulators, such as detoxifying and antimicrobial responses (see Additional file 10: Fig. S10B). Both programs did detect significant enrichment of categories related to extracellular material and cuticle collagens, the latter of which was previously found to be an enriched category of genes downregulated in *C. elegans* exposed to *Staphylococcus aureus* [40].

These data suggest that while ADR-1 RNA binding may be important for survival to *P. aeruginosa* infection, genes regulated by ADR-1 may not be those induced upon infection and perhaps could be altered even prior to infection. Consistent with this, using qRT-PCR and comparing to RNA isolated from wildtype animals grown under the same feeding conditions, all four genes downregulated in RNA isolated from *adr-1(-)* animals exposed to *P. aeruginosa* (Fig. 3C) were also significantly downregulated in *adr-1(-)* animals feeding on OP50 (Fig. 3F). In sum, these data indicate that RNA binding by ADR-1 regulates hundreds of genes during infection, which may be interesting for future studies to understand the importance of ADR-1 upregulation during infection. However, these data also indicate that these genes, and perhaps others that are regulated by both ADR-1 and ADR-2 and are important for organismal survival to infection, are misregulated prior to *P. aeruginosa* exposure.

### Worms lacking *adrs* exhibit decreased collagen expression

**Fig. 4:**
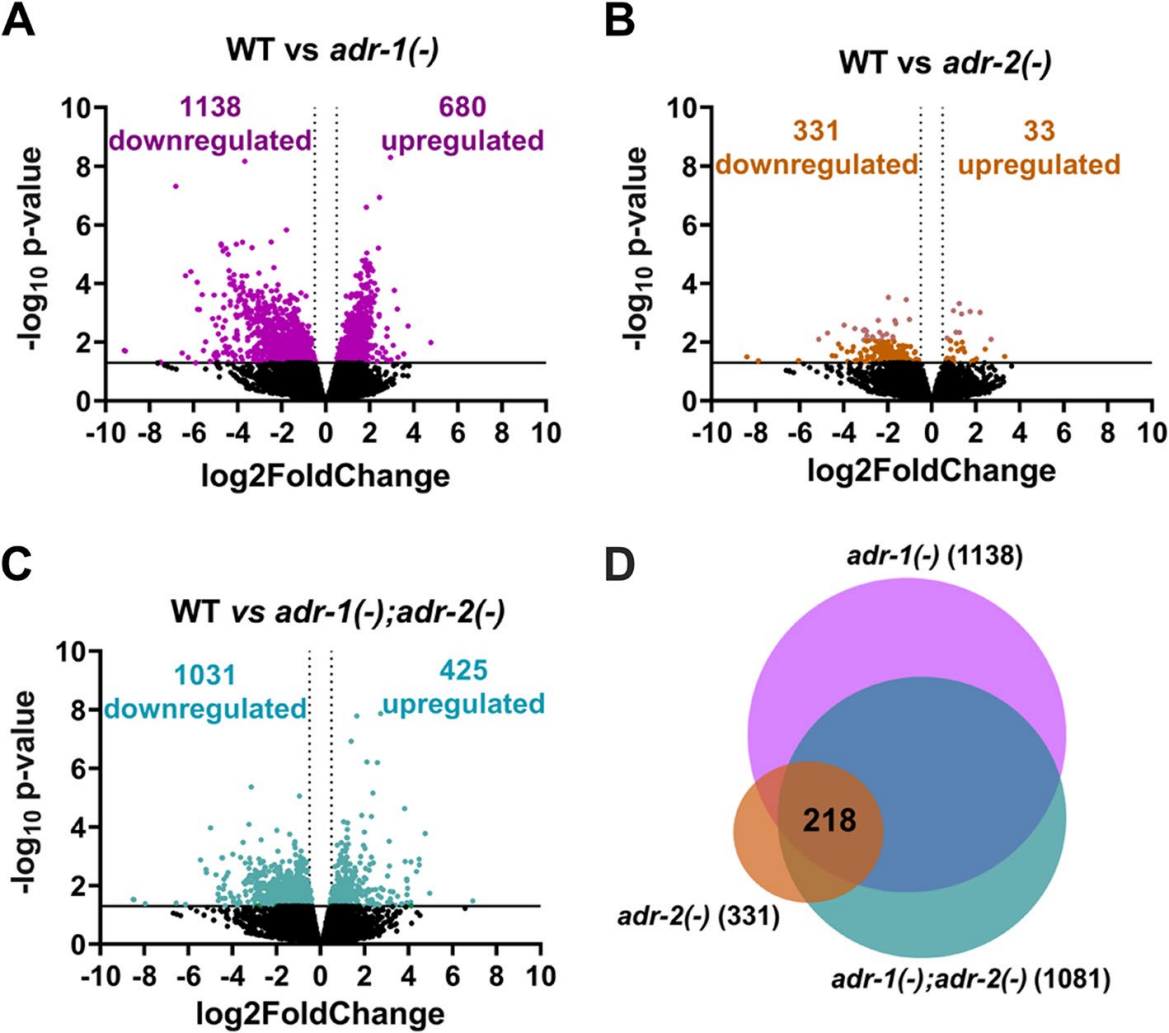
Altered gene expression in *adr* mutant animals. **A–C** Dots represent individual genes down- or upregulated or not significantly differentially expressed (black) in RNA-seq data from WT animals compared to *adr-1(-)* (purple) (A), *adr-1(-);adr-2(-)* (green) (**B**) and *adr-2(-)* (yellow) grown on OP50. Average log_2_ fold change (*x*-axis) is plotted against the negative log_10_ *p*-values (*y*-axis). Genes considered significantly differentially expressed exhibited log_2_ fold change of |0.5| (light gray dotted vertical lines) and* p* ≤ 0.05 [log_10_ *p*-value of 1.3, solid black horizontal line]. **D** Overlap of genes downregulated in (**A**, **B** and **C**)

To take an unbiased approach to understanding the role of ADARs in regulating basal expression of genes important for survival to *P. aeruginosa* infection, transcriptome-wide RNA sequencing was performed on RNA isolated from wildtype, *adr-1(-), adr-2(-)* and *adr-1(-);adr-2(-)* animals that were grown similar to the slow-killing assay, but exposed to only OP50 at 25 °C for 7 h. Polyadenylated RNA was isolated from three biological replicates of each genotype and subjected to high-throughput sequencing. Differential gene expression changes were analyzed in the wildtype RNA-seq dataset compared to the RNA-seq datasets of *adr-1(-), adr-2(-)* single mutant and the *adr-1(-);adr-2(-)* double mutant animals. The *adr-1(-)* and *adr-1(-);adr-2(-)* RNA-seq datasets had the largest number of significantly differentially expressed genes (*p* < 0.05, log_2_fold change >|0.5|) with over 1800 (Fig. 4A, Additional file 11: Table S11) and nearly 1500 (Fig. 4B, Additional file 12: Table S12) misregulated genes identified, respectively, whereas approximately 350 differentially expressed genes were identified in the *adr-2(-)* RNA-seq dataset (Fig. 4C, Additional file 13: Table S13). It is unclear why the RNA from the *adr-2(-)* animals exhibited less overall gene expression changes but does suggest that more genes may be affected by loss of *adr-1* than the complete loss of editing, which is consistent with our previous developmental assessment of ADR-1 and ADR-2 function [20].

To identify genes that might underlie the *adr* mutant animals’ enhanced susceptibility to *P. aeruginosa* infection, transcripts that were commonly misregulated across all three RNA-seq datasets were identified. Overlap of the upregulated transcripts in the *adr-1(-)* (680), *adr-2(-)* (33) and *adr-1(-);adr-2(-)* (425) RNA-seq datasets revealed only 4 commonly upregulated genes (*F01D4.8*, *Y116F11B.10*, *C17C3.3*, *F21C10.13*). However, overlap of the downregulated transcripts in the *adr-1(-)* (1138), *adr-2(-)* (331) and *adr-1(-);adr-2(-)* (1081) RNA-seq datasets revealed nearly 220 commonly downregulated genes (Fig. 4D, Additional file 14: Table S14). The genes regulated by both ADR-1 and ADR-2 had almost no overlap with genes regulated in response to *P. aeruginosa* infection (Dataset accession number: GSE5793) (2/218, Additional file 14: Table S14, *Staphylococcus aureus* infection (Dataset accession number: GSE2405) (0/218, Additional file 14: Table S14) or *Enterococcus faecalis* infection (Dataset accession number: GSE95636) (2/218, Additional file 14: Table S14). Using the WormCat software, GSEA revealed only one significantly enriched category—extracellular material (*p* value = 2.8*10^–07^) (Additional file 15: Fig. S15A). Further classification (category 2 output) of this enriched category revealed that 15 of the 17 misregulated genes associated with the extracellular material category were members of the collagen gene family (Additional file 15: Fig. S15B). Analysis with FuncAssociate also revealed significant enrichments for the collagen family and structural components of the cuticle (Additional file 15: Fig. S15C). Collagens are the major component of cuticle which is the outer surface of *C. elegans* and acts as a barrier between the animal and the environment [44]. Collagen expression was observed to be altered in *C. elegans* during recovery from acute *P. aeruginosa* infection [45], and early genetic studies demonstrated that loss of the cuticular collagen gene, *col-179*, led to enhanced susceptibility to *P. aeruginosa* infection [35]. Interestingly, *col-179* is one of the collagen genes in the downregulated transcripts present in our *adr-1(-)*, *adr-2(-)* and *adr-1(-);adr-2(-)* RNA-seq datasets from animals fed typical bacteria (*E. coli* OP50) (see Additional files 11, 12 and 13). To independently validate the changes in collagen gene expression, RNA was isolated from three independent biological replicates of the *adr* mutant strains grown as in the slow-killing assay but exposed to only OP50 and qRT-PCR was performed for three collagen genes, *col-179*, *col-106* and *col-135*. Consistent with the RNA-seq results, loss of either *adr-1* or *adr-2* or loss of both *adrs* resulted in a significantly decreased expression of the collagens when grown on OP50 (Additional file 16: Fig. S16A-C). To further explore the altered common *adr*-regulated genes, a second, independent analysis was performed using the extracellular specific software, Matrisome Annotator [46], which indicated that 14 of the 15 collagen genes were in fact cuticular collagens (Additional file 17: Table S17). Together, these data indicate that *adr* mutant animals have altered collagen expression and suggest that these molecular defects may impact cuticle structure and pathogen susceptibility.

### Worms lacking *adrs* exhibit altered cuticle structure and survival to several bacterial species

**Fig. 5:**
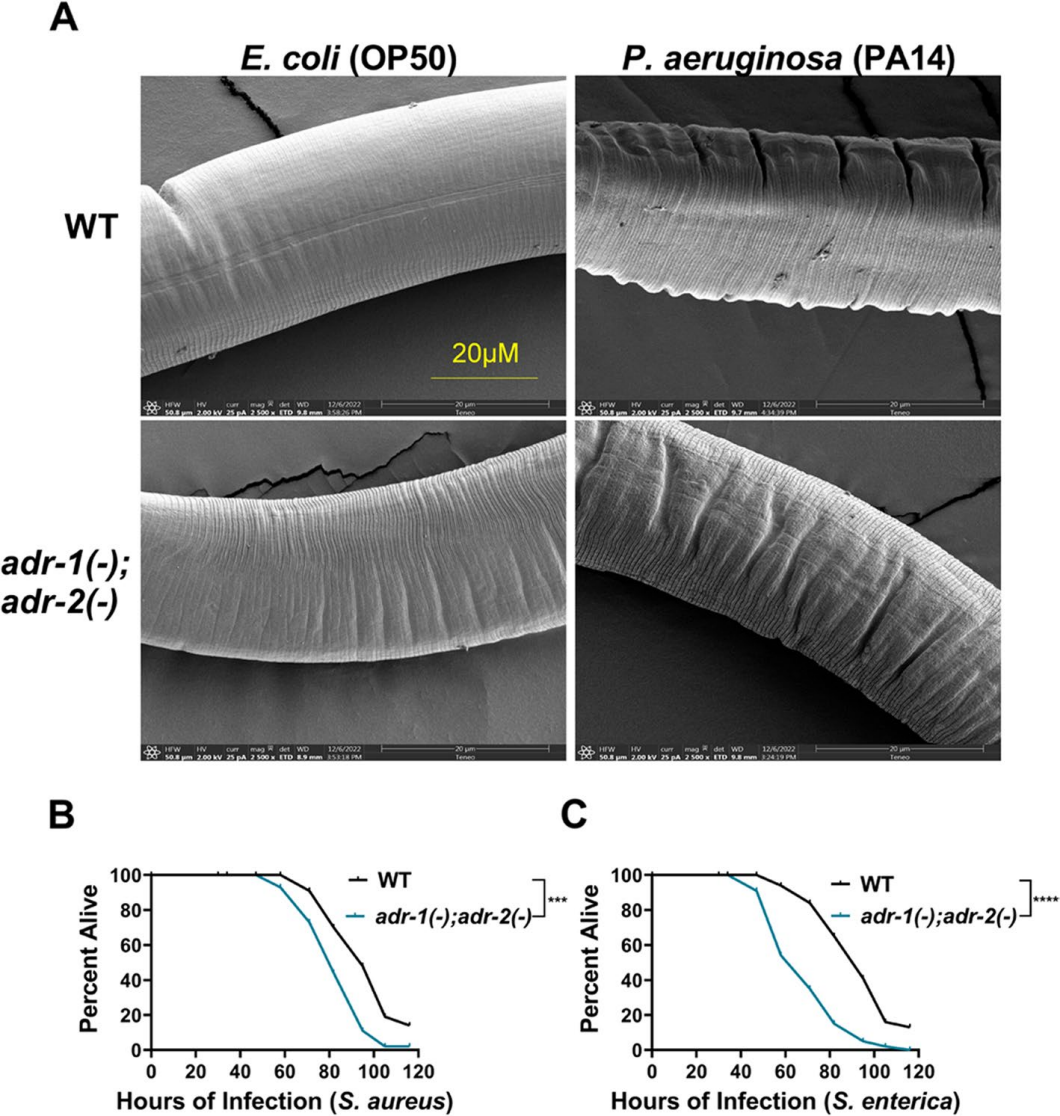
Loss of *adrs* results in altered cuticle structure and enhanced susceptibility to several pathogenic bacterial species. **A** Representative SEM images of the indicated strains after exposure to the bacterial strains indicated. Images were captured and categorized by blinded individuals (see methods) with 15 images available for wildtype animals fed OP50, 10 images available for wildtype animals exposed to PA14, 15 images available for *adr-1(-); adr-2(-)* animals fed OP50 and 10 *adr-1(-); adr-2(-)* animals exposed to PA14. Scale bar of image is 20 µm. **B**,** C** Representative survival curve (of three biological replicates) for the indicated animals subjected to the slow-killing assay and scored for survival in response to *S. aureus* (**B**) and *S. enterica* (**C**)*.* Statistical significance was determined using OASIS, ****p < 0.0001, ***p < 0.001. Survival assay biological replicates are provided in Additional file 18: Fig. S18A-D

As the molecular data suggests that *adr* mutant animals have decreased expression of collagen genes, the cuticles of wildtype and *adr-1(-);adr-2(-)* animals were analyzed by scanning electron microscopy (SEM) for gross ultrastructural defects. Both strains of animals were grown as in the slow-killing assay and then fed either *E. coli* (OP50) or *P. aeruginosa* (PA14) for 7 h. The cuticle of wildtype animals changed from smooth to wrinkled after *P. aeruginosa* exposure (Fig. 5A). Wrinkled cuticles are associated with the presence of a thinner hypodermis and/or alterations in the connections between the cuticle and hypodermis in aging animals [47]. This observation suggests that the cuticle structure changes in response to pathogen infection and has been previously observed in other SEM studies of wildtype *C. elegans* exposed to *P. aeruginosa* [48]. Interestingly, the cuticle of *adr-1(-);adr-2(-)* animals fed on *E. coli* appear to be more wrinkled compared to wildtype animals of same age and exposure conditions (Fig. 5A). The cuticle of *adr-1(-);adr-2(-)* animals exposed to *P. aeruginosa* was further wrinkled (Fig. 5A). However, the difference between the cuticles of wildtype and *adr-1(-);adr-2(-)* animals was less drastic in the *P. aeruginosa* exposure compared to the *E. coli* (OP50) exposure (Fig. 5A)*.* Together, these data suggest that ADARs regulate collagen levels, which in turn impacts cuticle structure and the ability of the animal to defend against pathogens.

As other mutant animals with altered cuticles have altered survival to a range of pathogens [48], we sought to examine the survival of *adr* mutant animals to two additional paradigmatic human pathogens: *Staphylococcus aureus* and *Salmonella enterica*. The gram-negative bacteria *S. enterica* can proliferate and establish infection in *C. elegans* [49, 50]. The gram-positive bacteria *S. aureus* has also previously been shown to both infect and kill *C. elegans* [51]*.* The standard slow-killing assay was performed with wildtype and *adr-1(*-*)*;*adr-2(-)* mutant animals on small lawns of *S. aureus* (Fig. 5B, Additional file 18: Fig. S18A, B) and *S. enterica* (Fig. 5C, Additional file 18: Fig. S18C, D). As expected, wildtype animals exposed to either *S. aureus or S. enterica* die over the course of several days (Fig. 5B, C). Survival of animals lacking both *adrs* was significantly shorter than wildtype animals when grown on either *S. aureus* (Fig. 5B) or *S. enterica* (Fig. 5C). Together, these data indicate the reproducible sensitivity of *adr* mutant animals to opportunistic human pathogens. Collectively, these data indicate that *C. elegans* ADARs can play important gene regulatory roles to contribute to the formation of physical barriers critical for promoting organismal survival to pathogen infection. Future research aimed at determining the susceptibility of ADAR mutants in other model systems as well as further mechanistic studies of how ADARs regulate collagen expression and the specific cuticular collagens that are key to organismal defense to infection are critical to improving our understanding of the complex relationship of ADARs and innate immunity.

## Discussion

In this study, we examined the contribution of *C. elegans* ADARs to survival from infection by opportunistic human pathogens. Specifically, we found that *adr* mutant animals are susceptible to both gram-negative (*Pseudomonas aeruginosa* and *Salmonella enterica*) and gram-positive (*Staphylococcus aureus*) bacteria. Using a combination of high-throughput sequencing, microscopy and functional genetics, we determined that ADR-1 and ADR-2 function together to regulate collagen expression, and the absence of these RNA-binding proteins results in altered cuticle structure, which in turn may render these animals more susceptible to infection.

At present, it is unknown how ADR-1 and ADR-2 regulate collagen expression. The ADAR family of RBPs can regulate gene expression in both editing-dependent and independent manners [52]. Our data indicates a role for ADR-1 RNA binding in regulating survival to pathogen infection (Fig. 3A) but does not eliminate the possibility of editing-dependent regulation, as ADR-1 binding to RNA has previously been shown to both promote and inhibit A-to-I editing by ADR-2 [21, 37]. Loss of *adr-1* leads to milder effects on editing compared to animals lacking *adr-2* or those lacking *adr-1* and *adr-2*, both of which completely lack editing. If survival to pathogen exposure was editing-dependent, the *adr-1(-)* animals would have an intermediate phenotype, similar to that observed for the chemotaxis defects of *adr* mutant animals [19]. Furthermore, from examination of six published manuscripts that perform unbiased RNA editing site identification in *C. elegans* [20, 21, 38, 53–55], (Dataset accession numbers: GSE110701, GSE151916, GSE51556, SRP028863, GSE83133, GSE98869) we did not observe A-to-I editing events of any of the misregulated collagen genes in our study (Additional file 17: Table S17). Interestingly, defects in RNA modification have been previously connected to altered collagen expression in *C. elegans* [56]. In the previous study, loss of methylation of cytosine (5mC) on ribosomal RNA (rRNA) resulted in decreased translation of cuticular collagen genes.

It is possible that ADARs are regulating collagen expression by directly binding to each of the misregulated collagen genes. In fact, two of the collagen genes, *col-179* and *col-106*, that exhibit decreased expression in the *adr* mutant animals were previously identified as ADR-1 bound mRNAs [20]. In addition, we also observed decreased expression of *col-179* and *col-106* in ADR-1 dsRBD1 mutant animals after exposure to *P. aeruginosa* (Additional file 9: Table S9). However, previous studies did not observe ADR-2 binding to these transcripts [37]. An alternative possibility is that ADR-1 and ADR-2 impact signaling pathways that control collagen expression, including potentially binding to and directly regulating expression of key transcription factors and kinases in these pathways. In particular, some of the cuticular collagens misexpressed upon loss of *C. elegans adrs* are regulated by the TGF-b (6/15 genes overlap) and/or insulin signaling (4/15 genes overlap) pathways [57, 58]. We have not observed misregulation of any of the canonical TGF-b pathway genes (*daf-1*, *daf-4*, *daf-8*, *daf-14*, *daf-3*, *daf-5*) in *adr* mutant animals in this study (Additional files 11, 12 and 13, Tables S11-13) or other tissue-specific studies [21]. However, for the insulin signaling pathway, we previously observed that the transcription factor which controls collagen expression, SKN-1, has reduced mRNA expression in the nervous system of *adr-2(-)* animals [21]. We did not observe altered *skn-1* expression in the RNA-seq analysis of young adult *adr* mutant animals presented in this work (Additional files 11, 12 and 13, Tables S11-13). Future work should explore whether changes in collagen-regulating pathways, such as those driven by SKN-1, are misregulated in the nervous system of *adr* mutant animals and whether this could contribute to altered cuticle structure. It has been proposed that although the epidermis plays a major role in synthesizing the cuticle, neurons can sense both the environment and tension to influence collagen dynamics [59]. In this regard, it was recently demonstrated that a neural G-protein coupled receptor, NPR-8, dynamically regulates collagen expression and cuticle structure in response to temperature changes and infection [48, 60]. Furthermore, loss of *npr-8* leads to increased resistance to pathogen infection [48]. As loss of *adrs* and *npr-8* have opposite phenotypes with respect to collagen expression and survival, these factors may be antagonistically regulating the same pathway. Experiments assessing pathogenic survival and cuticle morphology with animals that lack both *npr-8* and *adrs* would be an interesting future direction.

Regardless of the mechanism ADARs employ, it is unclear how changes in cuticle collagen expression and morphology can influence survival of *C. elegans* exposed to pathogenic bacteria, such as *P. aeruginosa*, which infect and colonize the intestine [26]. The epidermis and intestine are both epithelial tissues and use similar pathways to respond to pathogenic infection, including the master regulator of innate immunity, the p38 MAP kinase (MAPK) pathway [61]. Interestingly, a recent study did report that animals lacking *pmk-1*, the *C. elegans* p38 MAPK, had altered expression of collagens when grown in the presence of normal bacteria [62]. Lack of *pmk-1* does result in animals with enhanced susceptibility to *P. aeruginosa* infection [63]; however, the *pmk-1* phenotype is much more dramatic (approximately 80% of mutant animals die before wildtype animals begin to die) than lack of *adrs*. Furthermore, only two collagens (*col-62* and *col-135*) are commonly co-regulated in *adr* and *pmk-1* mutants (Dataset accession number: GSE192941) (Additional file 14: Table S14), and loss *adrs* and *pmk-1* have opposite effects on expression of these two collagen genes. These data also suggest that, while *adr* mutant animals exhibit decreased expression of several cuticle collagens, the enhanced susceptibility phenotype may not be a result of decreased expression of each individual collagen. Consistent with this, Sellegounder et al*.* [48] performed RNAi on seven individual collagens (*col-80*, *col-93*, *col-98*, *col-101*, *col-103*, *col-160* and *col-179*) and found only reduction in *col-101* and *col-179* resulted in enhanced susceptibility to *P. aeruginosa* infection.

While *col-179* was initially identified in one of the first screens for altered survival to *P. aeruginosa* infection [35], exactly how loss of a specific collagen impacts pathogenesis is unknown. With the recent identification of NPR-8 and now ADARs as regulators of *col-179* and cuticle morphology ([48] and this study), it is critical to delve further into the cellular processes in these mutants that impact pathogenesis. It is unlikely that these cuticle changes are impacting pathogen burden in the intestine and in support of this, we observed that both wildtype and *adr* mutant animals have comparable amounts of *P. aeruginosa* in the intestinal lumen (Additional file 19: Fig. S19). This is consistent with reports of *npr-8(-)* animals, which had decreased pathogen burden due to defecation defects; but upon restoration of proper defecation, *npr-8(-)* animals remained resistant to *P. aeruginosa* infection [48]. In addition, while early studies indicated that intestinal *P. aeruginosa* levels correlate with survival [26], the virulence from *P. aeruginosa* is multi-factorial [64]. Moreover, colonization of the intestine by other human pathogens, ex. *E. faeceium*, does not impact survival [65]. Future studies should focus on determining the critical tissues and pathways that regulate collagen expression to impact survival. It would be interesting to see if similar to pathogenic fungi, which colonize the epidermis, *P. aeruginosa* infection could also be impacted by antimicrobial peptide production by the MAPK and TGF-b pathways [66, 67]. It is also possible that other molecules, such as the recently identified meisosome signaling structures [68] or abundant intrinsically disordered proteins [69], could function to regulate survival to infection and aberrant cuticle morphology prevents proper function. An alternative possibility is that the stiffness or other mechanical properties of the cuticle impact survival via an unknown mechanism, such has recently been shown for mate recognition [70]. Interestingly, it has also recently been shown that the elasticity and strength of the *C. elegans* cuticle changes with age of adult animals [71]. Age of animals was also one of the first differences reported to impact survival from *P. aeruginosa* infection over 20 years ago [26]; however, the causes of the differential survival of L4 and adult animals has not been defined.

It is also important to note that, in our study, animals were grown at 25ºC prior to isolating RNA for high-throughput sequencing or SEM imaging, which could influence cuticular structure. Previous studies have shown that the primary transcriptional regulator of cellular response to elevated temperature, HSF-1, is a major regulator of collagen gene expression both in the presence and absence of heat shock [72]. In total, comparing genes misregulated in *adr* mutant animals (this study) and animals lacking *hsf-1* [72] (Dataset accession number: SRP078295), we observed 10 of the 15 collagen genes were commonly misregulated. Similar to *skn-1*, we do not observe altered *hsf-1* mRNA expression in the RNA isolated from *adr* mutant animals, but *hsf-1* expression was previously observed to be downregulated in the nervous system of *adr-2(-)* animals [21]. Future experiments should aim to dissect how temperature differentially impacts the cuticular structure of *adr* mutant animals and if HSF-1 is important for regulation of collagen gene expression by ADARs. Importantly, our data does indicate that despite affecting cuticle morphology, the lack of ADARs does not appear to make animals generally sensitive to acute stress. In fact, our data indicate that *adr* mutant animals survive acute heat (35 °C) stress significantly better than wildtype animals (Additional file 3: Fig. S3). Interestingly, in studying the impact of ADARs in the nervous system, a recent publication from our lab demonstrated that larval animals lacking *adr-2* also survive hypoxia induced by cobalt chloride significantly better than wildtype animals [73]. Together, these data indicate that ADARs can impact a variety of pathways to both promote and inhibit resistance to various stressors and suggest that, while it is possible that cuticle defects can lead to sensitivity to a number of stressors, there are also pathogen-specific signatures that can lead to increased susceptibility to infection.

In addition to ADR-1 and ADR-2 functioning together to regulate collagen gene expression and organismal resistance to pathogen infection, our study revealed hundreds of transcripts that are regulated by ADR-1 binding upon exposure to pathogen. Interestingly, we also see an increase in ADR-1 protein expression upon pathogen exposure, which raises the possibility that ADR-1 could be binding to new targets in response to infection and potentially has additional functions beyond promoting survival to infection. Recently, roles for RNA-binding proteins and small RNAs in promoting pathogenic memory and transgenerational inheritance have been identified [74, 75]. Future studies should explore changes in ADR-1 binding targets in response to infection and their effects on immunological memory.

## Conclusions

This study revealed a critical role of the *C. elegans* ADAR family of RNA-binding proteins in promoting cuticular collagen expression and defense from pathogenic microbes. Previous studies of this RNA-binding protein family have suggested a role in the antiviral response, but our data indicate a broader function of ADARs in innate immunity. This work sets the stage for future studies aimed at mechanistic dissection of how ADARs control collagen expression and the tissue-specific roles these proteins play in innate immunity. In addition, our study provides a list of targets regulated by ADR-1 RNA binding which could be critical for future research on ADAR function in immunity and development.

## Methods

### Worm strains and maintenance

All worms were maintained under standard laboratory conditions on nematode growth media (NGM) seeded with *Escherichia coli* OP50. Worm strains used in this study and previously published are wildtype (N2), BB19 *adr-1(tm668)*, BB20 *adr-2(ok735)*, BB21 *adr-1(tm668);adr-2(ok735)* [76], BB2 *adr-1(gv6)*, BB3 *adr-2(gv42)* [19], BB19 *adr-1(tm668)* + blmEx1[3XFLAG-*adr-1 genomic, rab3::gfp::unc-54*]) BB21 *adr-1(tm668);adr-2(ok735)* + blmEx1[3xFLAG-*adr-1* genomic, *rab3::gfp::unc-54*]) [38]. BB21 *adr-1(tm668)* + *blmEx11*[*3XFLAG-adr-1* genomic with mutations in *dsRBD1 (K223E, K224A, and K227A*), *rab3::gfp::unc-54 (3*′ *UTR)*] [37]. Additional strains used in this study were *adr-2(uu28)* (a kind gift from Brenda Bass) and the newly generated ALM63 *adr-1(tcn1)* strain and the HAH36 V5-ADR-1; 3xFLAG-ADR-2 strain, which were created by CRISPR using the large deletion protocol [77] and [78], respectively. Guides and repair templates are listed Additional file 20: Table S20 (IDT). For ALM63, the injection mix contained 25 µM KCl, 7.5 mM HEPES, pH 7.4, 4.9 µM Cas9 (Invitrogen, TrueCut), 5 µM tracrRNA (IDT), 2 µM *dpy-10* crRNA, 25 µM each of two crRNAs to *adr-1*, 2 µM *dpy-10* single-stranded oligo nucleotide (ssODN) repair sequence and 5 µM of a target ssODN to *adr-1*. To avoid compounding effects from off-target mutations, the generated ALM63 strain was crossed twice with the wildtype strain. For HAH36, the V5 epitope at the N-terminus of ADR-1 and 3 copies of the FLAG epitope at the N-terminus of ADR-2 were constructed in wildtype worms individually, back-crossed to wildtype worms and then crossed to generate HAH36. Injection mix for the V5-ADR-1 and 3xFLAG-ADR-2 strains included 1.5 µM Cas9 (IDT, Alt-R Cas9 nuclease V3), 4 µM tracrRNA (IDT), 4 µM of crRNA (IDT), 37 ng/µl *rol-6* plasmid (HAH293) and 1 µM target ssODN. Genomic modifications were verified using PCR (primers listed in Additional file 20: Table S20) and Sanger sequencing. Western blotting was also performed to verify the V5 and 3xFLAG insertions.

### Pathogenic bacterial growth

Three pathogenic bacterial strains were used: *P. aeruginosa* PA14 (a kind gift of Read Pukkila-Worley), *S. enterica* SL1344 and *S. aureus* MSSA476 from (kind gifts of Jingru Sun, Washington State University). Bacterial strains were freshly streaked on LB plates and grown as 5 ml cultures at 37 °C overnight. The next day, 20 μl of culture was spotted onto 6 cm NGM agar plates. Plates were incubated at 37 °C overnight (not exceeding 16 h) and then moved to 25 °C for at least 24 h before starting the slow-killing assay.

### Slow-killing assay

Slow-killing assays were performed as previously described [26, 48] with slight modifications. For each assay, 45 worms of each strain were plated on each of three NGM plates containing 0.05 mg/ml 5-Fluoro-2´-deoxyuridine (MP Biomedical) spotted with 20 µl of a given bacterial strain (grown as described above). Plates were incubated at 25 °C and after 24 h, animals were scored as dead or alive at least once every 11–13 h over the course of 120 h.

### *P. aeruginosa* exposure assay for gene expression

Gravid adult worms were collected in 1 × M9 buffer (3 g KH_2_PO_4_, 6 g Na_2_HPO_4_, 5 g NaCl, 1 ml 1 M MgSO_4_, H_2_O to 1 L) and incubated with 0.5 M NaOH in 1.2% NaClO (Fisher) to release eggs. Eggs were washed thoroughly with 1 × M9 buffer and hatched overnight at 20˚C to obtain synchronized first larval stage (L1) animals. Hatched L1 animals were washed with 1 × M9 and grown at 20 °C on standard NGM plates with OP50 for 42 h. For exposures of each strain, three 10 cm NGM plates were seeded with 40 µl of OP50 or PA14. After exposure for 7 h at 25 °C, worms were washed with 1 × M9 buffer and collected in TRIzol (Invitrogen).

### Pharyngeal pumping rate assay

These experiments were performed as previously described [79]. Briefly, 6-cm NGM plates were seeded with 30 µl of an overnight culture of *P. aeruginosa* (PA14) and incubated as described earlier. Fifteen synchronized young adult worms were transferred to the seeded plates and incubated 24 h at 25 °C. Individual worms were tracked under Carl Zeiss Stemi 305 microscope, and the number of contractions of the pharyngeal bulb was counted over 30 s.

### RNA isolation and quantitative real-time PCR (qRT-PCR)

Total RNA was isolated using TRIzol (Invitrogen) and purified using TURBO DNase (Ambion) followed by the RNeasy Extraction kit (Qiagen). For qRT-PCR, 2 µg of DNase-treated RNA was subjected to cDNA synthesis using Superscript III (Invitrogen) reverse transcriptase and a combination of both random hexamers and oligo dT primers (Fisher Scientific). After reverse transcription, 20 µl of water was added to each cDNA sample. Gene expression was determined by qRT-PCR using SybrFast Master Mix (KAPA) and gene-specific primers using a Thermofisher Quantstudio 3 instrument. Primers for qRT-PCR (see Additional file 20: Table S20) were designed to span an exon-exon boundary. For each gene analyzed, a standard curve was generated using tenfold serial dilutions of the amplicon to test the relative concentration versus the threshold of amplification. Standard curves were plotted on a logarithmic scale in relation to concentration and fit of the line (r^2^ value). The r^2^ value was typically 0.99, and all data points fell within the standard curve. For each sample, cDNA concentration was measured in triplicate and three biological replicates were performed for each experiment.

### Western analysis

Synchronized L4 animals after exposure to either *P. aeruginosa* (PA14) or *E. coli* (OP50) at 25 °C for 7 h were collected in 1 × M9 buffer and washed 3 times. Collected animals were rocked for 20 min at room temperature. After a brief centrifugation step, the animals were pelleted, resuspended in 1 × SDS buffer (2% SDS, 50 mM Tris HCl, and 10% Glycerol) and snap-frozen using liquid nitrogen. Lysates were prepared by boiling the pellet for 15 min and vortexing every 7–8 min. Protein concentration was measured using Bradford reagent (Sigma) and then 100 mM DTT and bromophenol blue (0.1%) were added to the lysate before boiling for 5 min. An equivalent amount of protein lysate was subjected to SDS-PAGE and immunoblotting with antibodies against FLAG (Sigma), V5 (Cell Signaling), and β-actin (Cell Signaling).

### Library preparation, RNA sequencing, and analysis

DNase-treated RNA was incubated with oligo(dT) beads (Invitrogen) and followed by library preparation using a stranded RNA-seq library preparation kit (KAPA) as per the manufacturer’s instructions. Libraries were sequenced for SE75 cycles on an Illumina NextSeq 500 instrument at the Center for Genomics and Bioinformatics at Indiana University. The sequences obtained were run through FASTQC (version 0.11.9) to evaluate the quality of the sequencing reads. The summary of the sequences obtained, and the number of sequences flagged as low quality has been summarized in Additional file 21: Table S21. Single-stranded sequencing reads were aligned to *C. elegans* genome (WS275) using STAR (v2.7.6a) using the following parameters: outFilterMultimapNmax 1 \ outFilterScoreMinOverLread 0.66 \ outFilterMismatchNmax 10 \ outFilterMismatchNoverLmax 0.3. Uniquely mapped reads were then used as an input for running featureCounts (v2.0.1). The raw read counts obtained from featureCounts were used for differential gene expression analysis using DeSeq2. R studio version 4.1.1 was used to install Bioconductor package (v3.10) to load DeSeq2.

### Gene set enrichment, annotations and overlaps

Ontology enrichment analysis was performed by entering wormbase IDs in WormCat 2.0 [42] and FuncAssociate [43]. Matrisome annotator [46] was used to classify extracellular matrix genes. Overlaps between published datasets and this study were performed using either the VLOOKUP command in Excel or BioVenn [80], a web application for visualization of area proportional Venn diagrams.

### Scanning electron microscopy

Scanning electron microscopy (SEM) was performed as previously described [48]. Briefly, synchronized young adult animals were exposed to *P. aeruginosa* (PA14) or *E. coli* (OP50) for 7 h at 25 °C. Animals were removed from plates with 1 × M9 buffer, washed five times wherein the animals were allowed to settle by gravity. After washing, genotypes and exposures were blinded. Animals were incubated overnight in fixation buffer (2.5% glutaraldehyde, 1.0% paraformaldehyde, and 0.1 M sodium phosphate (Electron Microscopy Sciences)) at 4 °C. From this point forward, SEM image preparation and capture were done in the IU Center for Electron Microscopy by an SEM specialist (samples were blinded). Samples were then washed with 0.1 M sodium phosphate, and the sample suspension was placed in BEEM capsules (size 00) (Ted Pella, Inc.). Samples were dehydrated at room temperature in a graded series of ethanol (30%, 50%, 75%, 90%, 95%, and 100%) with incubation for 10 min at each step. Dried samples were placed in aluminum SEM stubs (Electron Microscopy Sciences); which were sputter coated at 45 nm with a Safematic CCU-010. The sputter coated target was composed of gold:palladium (80:20). SEM imaging was done using a ThermoFisher Teneo instrument set to 2.0 kV. Using all SEM images for a given strain (blinded), gross ultrastructure of the cuticle was categorized in terms of appearance using published SEM images of wildtype animals grown in normal bacteria [44, 48] and exposed to *P. aeruginosa* [48]. After independent categorization by three lab members, genotypes were revealed.

### Chemotaxis assay

A chemotaxis assay was performed with wildtype animals and those with a V5 epitope at the N-terminus of ADR-1 and three copies of the FLAG epitope at the N-terminus of ADR-2 (HAH36) as previously described [19]. Briefly, chemotaxis plates (10 cm) were spotted with 1 μl of butanone (1:1000 dilution in ethanol) and 1 μl of ethanol (control) equidistant from the midpoint of the chemotaxis plate. To anesthetize animals reaching these regions, 1 μl of sodium azide (1 M) was added to the attractant and control spots. Between 100 and 150 young adult animals were placed in a circle at the center of the plate. After 1 h, animals were counted to calculate a chemotaxis index. Chemotaxis index = animals at the attractant-animals at control)/total number of animals on the plates.

### RNA editing assay

Mixed stage worms were stored in TRIzol (Invitrogen) and RNA was extracted according to the methods described above. After DNase treatment and clean-up, the RNA was reverse transcribed with a gene-specific primer (Additional file 20: Table S20) using Superscript III (Invitrogen). The resulting cDNA was amplified by PCR with Phusion DNA polymerase (NEB) (primers listed in Additional file 20: Table S20). To confirm the resulting products were amplified from the RNA, negative controls were performed wherein the reverse transcription reaction was followed but without the addition of Superscript III. The resulting cDNA for *lam-2* gene was purified with gel electrophoresis and sent for Sanger sequencing (QuintaraBio).

### Quantification of intestinal bacterial load

Quantification of colony-forming units (CFU) was performed as previously described [33, 81], with slight modifications. Briefly, synchronized L4 staged animals were transferred to plates seeded with *P. aeruginosa* (samples were blinded). To distinguish colonies formed from the *P. aeruginosa* exposure rather than possible, residual *E. coli* from earlier developmental growth on OP50, a modified *P. aeruginosa* with resistance to Kanamycin was used in these assays (PA14::GFP). After 24 h at 25 °C, animals were washed with 1 × M9 buffer. To further eliminate bacteria that was attached to the animals and not within the intestine, washed animals were transferred to an unseeded NGM plate. After 15 to 20 min, 50 animals per condition were transferred to a 1.5-mL sterile tube containing 250 μL PBS with 0.1% Triton and lysed with a sterile pestle. Serial dilutions of the lysate were made, and the 10^−3^ dilution was spread onto LB/Kanamycin plates for the selective growth of *P. aeruginosa*. After 24 h at 37 °C, bacterial colonies were counted. Each colony represents a single cell. The amount of CFUs was calculated using the following formula: $CFU\;per\;worm=\left(\frac{Number\;of\;clories\;per\;plate\times10^{dilution\;factor}\times Plated\;volume\left(mL\right)}{Number\;of\;worms}\right)$. 

### Acute heat stress assay

The acute heat stress assay was performed as previously described [28]. All strains used were bleached to synchronize animals (as described in main text), which were then grown on normal NGM media at 20 °C to the L4 stage. Briefly, 45–50 L4 animals of WT, *adr -1(tm668)*, *adr-2(ok735)* and *adr-1(tm668); adr-2(ok735)* mutant strains were transferred to 6-cm OP50 seeded plates. Each strain was plated in triplicates. The plates were then sealed with parafilm and placed inside a plastic bag. Heat stress was performed by submerging the plates in a water bath maintained at 35 °C for 6 h. After 6 h, the animals were recovered at 20 °C for 14 h before live and dead animals were counted. All strains were assayed in three biological replicates.

### Supplementary Information

**Additional file 1: Fig. S1.** ADAR mutant animals are susceptible to *Pseudomonas* infection. (Related to Fig. 1) Survival curves of independent biological replicates for the ADAR mutant animals subjected to the slow-killing assay and scored for survival in response to *P. aeruginosa* strain (PA14) for Fig. 1A-D.

**Additional file 2: Fig. S2.** ADAR mutant worms do not exhibit enhanced susceptibility when grown in the presence of OP50. Survival curves of three independent biological replicates for the indicated animals subjected to the slow-killing assay and scored for survival in response to *P. aeruginosa* (PA14) and *E. coli* (OP50).

**Additional file 3: Fig. S3.** *adr* mutant animals are not sensitive to acute heat stress. Average percentage (%) of animals alive after acute heat stress for three independent biological replicates.

**Additional file 4: Fig. S4.** ADR-1 alone is not sufficient to rescue the susceptibility phenotype of the *adr-1(-);adr-2(-)* animals. Survival curves of all three independent biological replicates subjected to the slow-killing assay in *P. aeruginosa* strain (PA14).

**Additional file 5: Fig. S5.** The presence of epitope tags does not significantly alter chemotaxis behavior or RNA editing. Data from three independent biological replicates of the chemotaxis assay and Sanger sequencing analysis.

**Additional file 6: Fig. S6.** ADR-1 protein expression increases upon *P. aeruginosa* (PA14) exposure. (Related Fig. 2). Uncropped western blots of all biological replicates.

**Additional file 7: Fig. S7.** ADR-1 RNA binding is required for survival to *P. aeruginosa.* Survival curves of independent biological replicates for ADR-1 RNA binding mutant animals subjected to the slow-killing assay.

**Additional file 8: Table S8.** Differentially expressed genes in *adr-1(-)* animals compared to wildtype animals grown on PA14 for 7 h (Related to Fig. 3). Differentially expressed genes are listed as Wormbase annotations and Gene name.

**Additional file 9: Table S9.** Differentially expressed genes in ADR-1 dsRBD1 mutant animals compared to wildtype animals grown in grown on PA14 for 7 h (Related to Fig. 3). Differentially expressed genes are listed as Wormbase annotations and Gene name.

**Additional file 10: Fig. S10.** Gene set enrichment analysis of 332 misregulated genes in *adr-1(-)* animals and ADR-1 dsRBD1 mutant animals exposed to PA14. Gene set enrichment analysis using WormCat output and FuncAssociate output.

**Additional file 11: Table S11.** Differentially expressed genes in *adr-1(-)* animals compared to wildtype animals grown on OP50 for 7 h (Related to Fig. 4). Genes listed as Wormbase annotations for the three biological replicates.

**Additional file 12: Table S12.** Differentially expressed genes in *adr-1(-);adr-2(-)* animals compared to wildtype animals grown on OP50 for 7 h (Related to Fig. 4). Genes listed as Wormbase annotations for the three biological replicates.

**Additional file 13: Table S13.** Differentially expressed genes in *adr-2(-)* animals compared to wildtype animals grown on OP50 for 7 h (Related to Fig. 4). Genes are listed as Wormbase annotations for the three biological replicates.

**Additional file 14: Table S14.** Common downregulated genes in *adr-1(-), adr-2(-) and adr-1(-);adr-2(-*) animals compared to wildtype animals grown on OP50 for 7 h (Related to Fig. 4). Downregulated genes from Supplemental Table 11,12, and 13 were overlapped to find common differentially expressed genes.

**Additional file 15: Fig. S15.** Enrichment analysis of common downregulated genes in *adr-1(-), adr-2(-)* and *adr-1(-);adr-2(-)* grown on OP50. Enrichment analysis was done using wormcat and FuncAssociate for common downregulated genes.

**Additional file 16: Fig. S16.** qRT-PCR validation of downregulated collagen genes from RNA-seq analysis. qRT-PCR quantification of the level of the collagen genes in *adr* mutant animals.

**Additional file 17: Table S17.** Identification of cuticle collagen genes. The cuticle collagen genes identified from the Matrisome Annonatator software and editing site analysis.

**Additional file 18: Fig. S18.** Loss of *adrs* results in enhanced susceptibility to several pathogenic bacterial species. (Related to Fig. 5). Additional survival curves for the *adr-1(-);adr-2(-) animals* subjected to the slow-killing assay with *S. aureus* and *S. enterica.*

**Additional file 19: Fig. S19.** *P. aeruginosa* bacterial load is comparable in wildtype and *adr* mutant animals. PA14 bacterial load quantified for wildtype and *adr* mutant animals.

**Additional file 20: Table S20.** List of primers used in the study.

**Additional file 21: Table 21.** Summary of FASTQC analysis of the samples used for RNA-seq.

## Data Availability

All data generated or analyzed during this study are included in this published article, its supplementary information files and publicly available repositories. All bacterial and nematode strains can be provided upon request. Raw high-throughput sequencing reads and the full FASTQC reports have been uploaded to GEO under GSE223919.

## Abbreviations

ADAR: Adenosine deaminase that act on RNA
dsRBD1: Double-stranded RNA Binding Domain 1
qRT-PCR: Quantitative Real-Time PCR
CRISPR: Clustered regularly interspaced short palindromic repeats
STAR: Spliced Transcripts Alignment to a Reference
GEO: Gene Expression Omnibus
BEEM: Ballistic Electron Emission Microscopy
OASIS: Online Application for Survival Analysis
rRNA: Ribosomal RNA
