# Supplementary material for: ADARs regulate cuticle collagen expression and promote survival to pathogen infection
Source: BMC Biol. 2024 Feb 16;22:37. doi: 10.1186/s12915-024-01840-1 (PMC10870475; doi:10.1186/s12915-024-01840-1)
Supplement: Supplementary file 1 — Additional file 1: Fig. S1. ADAR mutant animals are susceptible to Pseudomonas infection. (Related to Fig. 1) Survival curves of independent biological replicates for the ADAR mutant animals subjected to the slow-killing assay and scored for survival in response to P. aeruginosa strain (PA14) for Fig. 1A-D. [file 12915_2024_1840_MOESM1_ESM.pptx]

## Slide 1
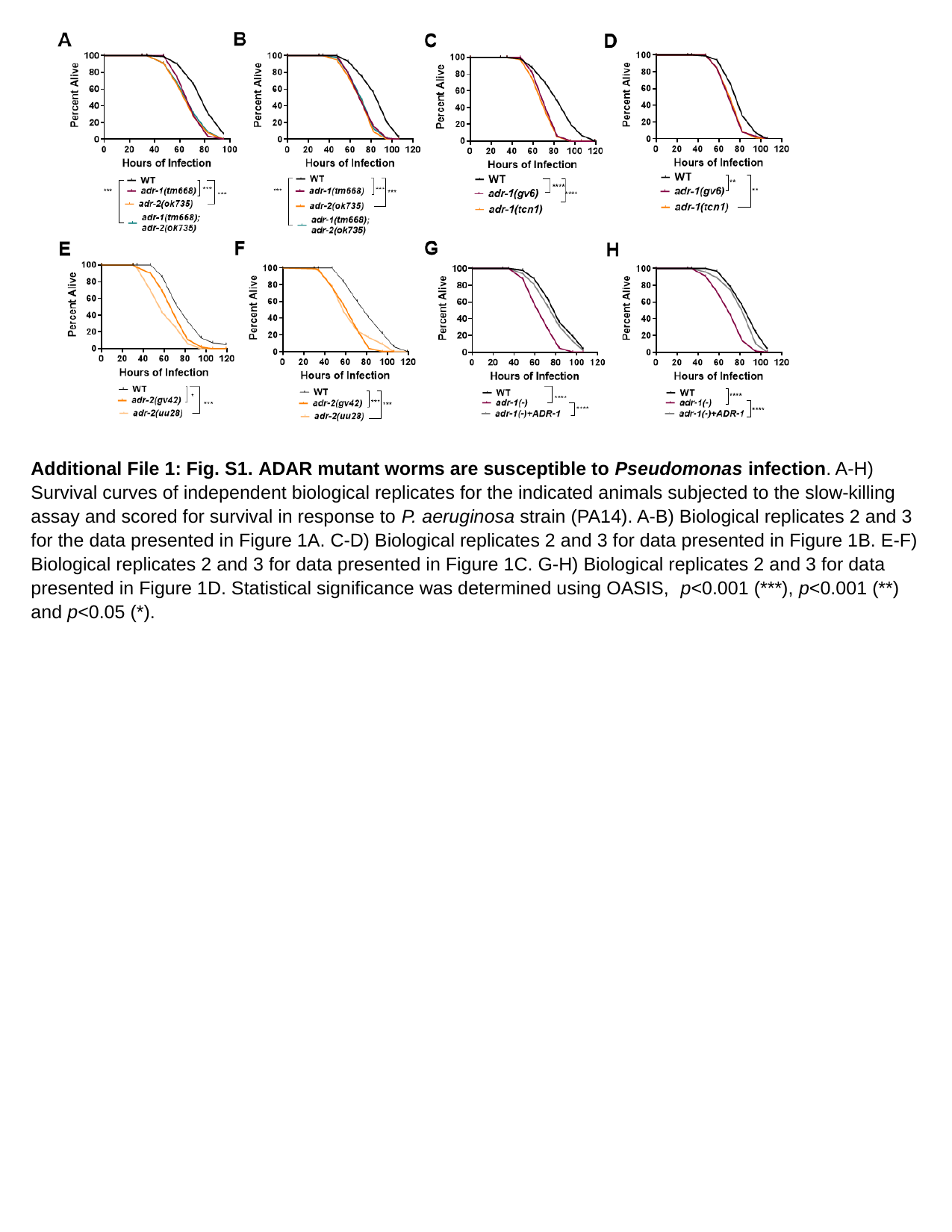

Additional File 1: Fig. S1. ADAR mutant worms are susceptible to Pseudomonas infection. A-H) Survival curves of independent biological replicates for the indicated animals subjected to the slow-killing assay and scored for survival in response to P. aeruginosa strain (PA14). A-B) Biological replicates 2 and 3 for the data presented in Figure 1A. C-D) Biological replicates 2 and 3 for data presented in Figure 1B. E-F) Biological replicates 2 and 3 for data presented in Figure 1C. G-H) Biological replicates 2 and 3 for data presented in Figure 1D. Statistical significance was determined using OASIS, p<0.001 (***), p<0.001 (**) and p<0.05 (*).
