# Supplementary material for: ADARs regulate cuticle collagen expression and promote survival to pathogen infection
Source: BMC Biol. 2024 Feb 16;22:37. doi: 10.1186/s12915-024-01840-1 (PMC10870475; doi:10.1186/s12915-024-01840-1)
Supplement: Supplementary file 2 — Additional file 2: Fig. S2. ADAR mutant worms do not exhibit enhanced susceptibility when grown in the presence of OP50. Survival curves of three independent biological replicates for the indicated animals subjected to the slow-killing assay and scored for survival in response to P. aeruginosa (PA14) and E. coli (OP50). [file 12915_2024_1840_MOESM2_ESM.pptx]

## Slide 1
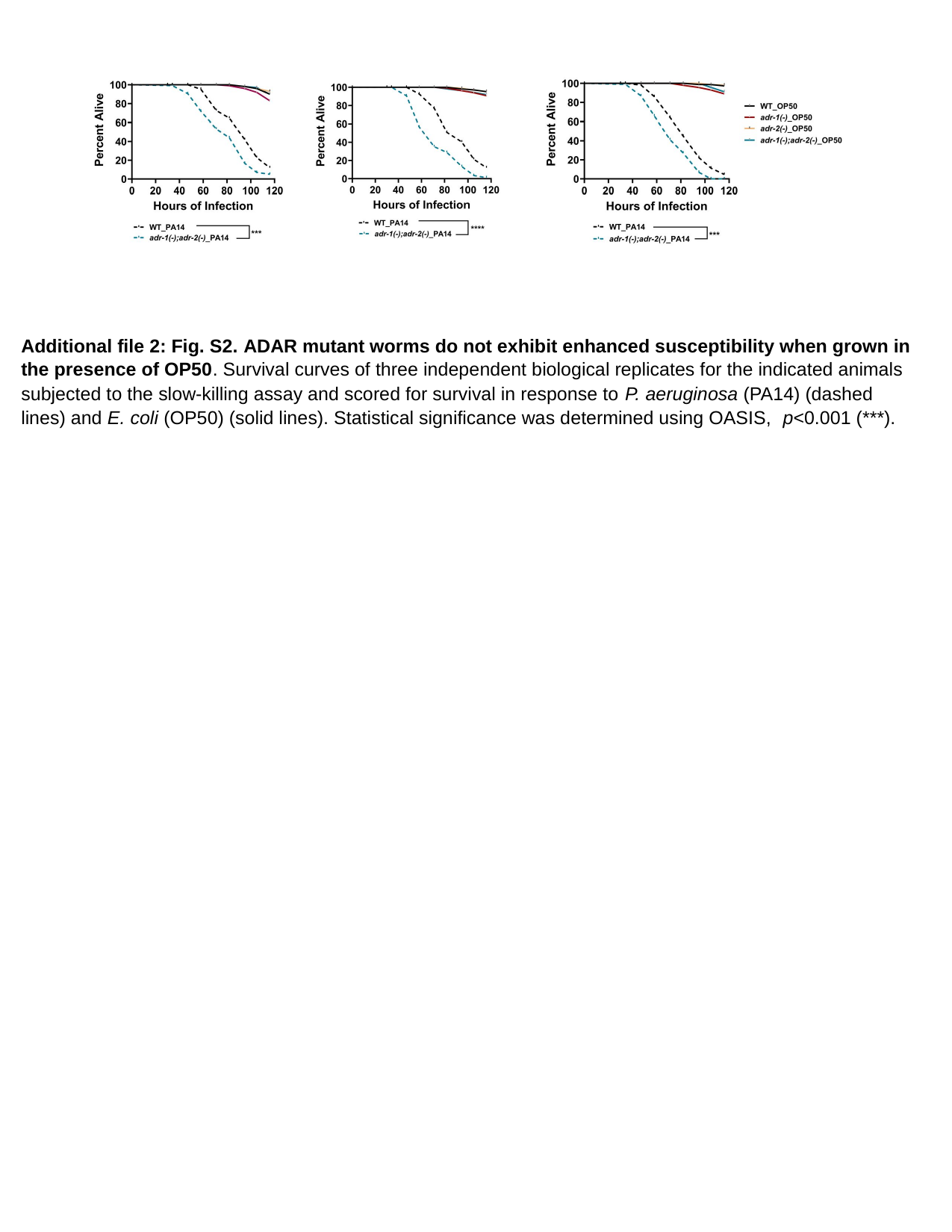

Additional file 2: Fig. S2. ADAR mutant worms do not exhibit enhanced susceptibility when grown in the presence of OP50. Survival curves of three independent biological replicates for the indicated animals subjected to the slow-killing assay and scored for survival in response to P. aeruginosa (PA14) (dashed lines) and E. coli (OP50) (solid lines). Statistical significance was determined using OASIS,  p<0.001 (***).
