# Supplementary material for: ADARs regulate cuticle collagen expression and promote survival to pathogen infection
Source: BMC Biol. 2024 Feb 16;22:37. doi: 10.1186/s12915-024-01840-1 (PMC10870475; doi:10.1186/s12915-024-01840-1)
Supplement: Supplementary file 3 — Additional file 3: Fig. S3. adr mutant animals are not sensitive to acute heat stress. Average percentage (%) of animals alive after acute heat stress for three independent biological replicates. [file 12915_2024_1840_MOESM3_ESM.pptx]

## Slide 1
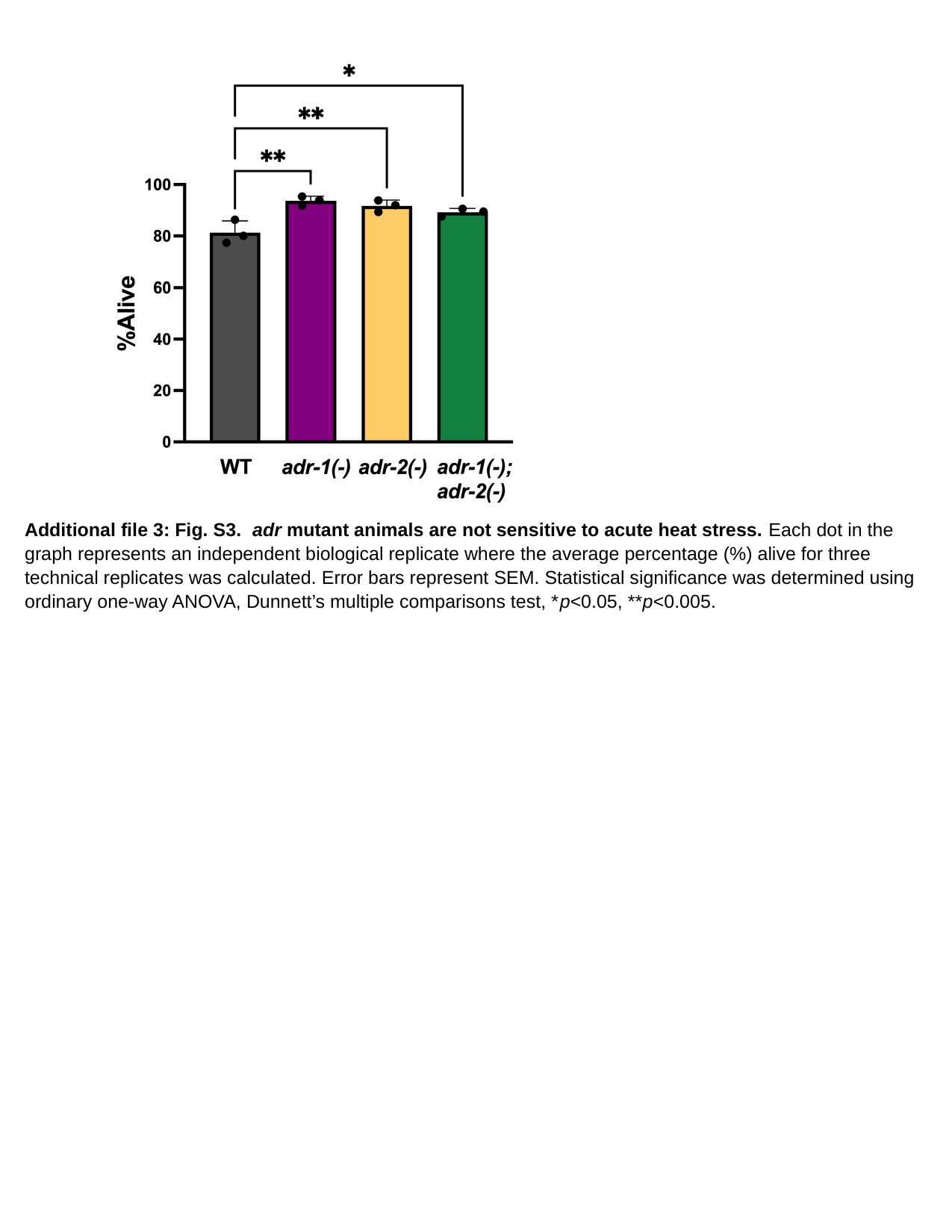

Additional file 3: Fig. S3.  adr mutant animals are not sensitive to acute heat stress. Each dot in the graph represents an independent biological replicate where the average percentage (%) alive for three technical replicates was calculated. Error bars represent SEM. Statistical significance was determined using ordinary one-way ANOVA, Dunnett’s multiple comparisons test, *p<0.05, **p<0.005.
