# Supplementary material for: ADARs regulate cuticle collagen expression and promote survival to pathogen infection
Source: BMC Biol. 2024 Feb 16;22:37. doi: 10.1186/s12915-024-01840-1 (PMC10870475; doi:10.1186/s12915-024-01840-1)
Supplement: Supplementary file 4 — Additional file 4: Fig. S4. ADR-1 alone is not sufficient to rescue the susceptibility phenotype of the adr-1(-);adr-2(-) animals. Survival curves of all three independent biological replicates subjected to the slow-killing assay in P. aeruginosa strain (PA14). [file 12915_2024_1840_MOESM4_ESM.pptx]

## Slide 1
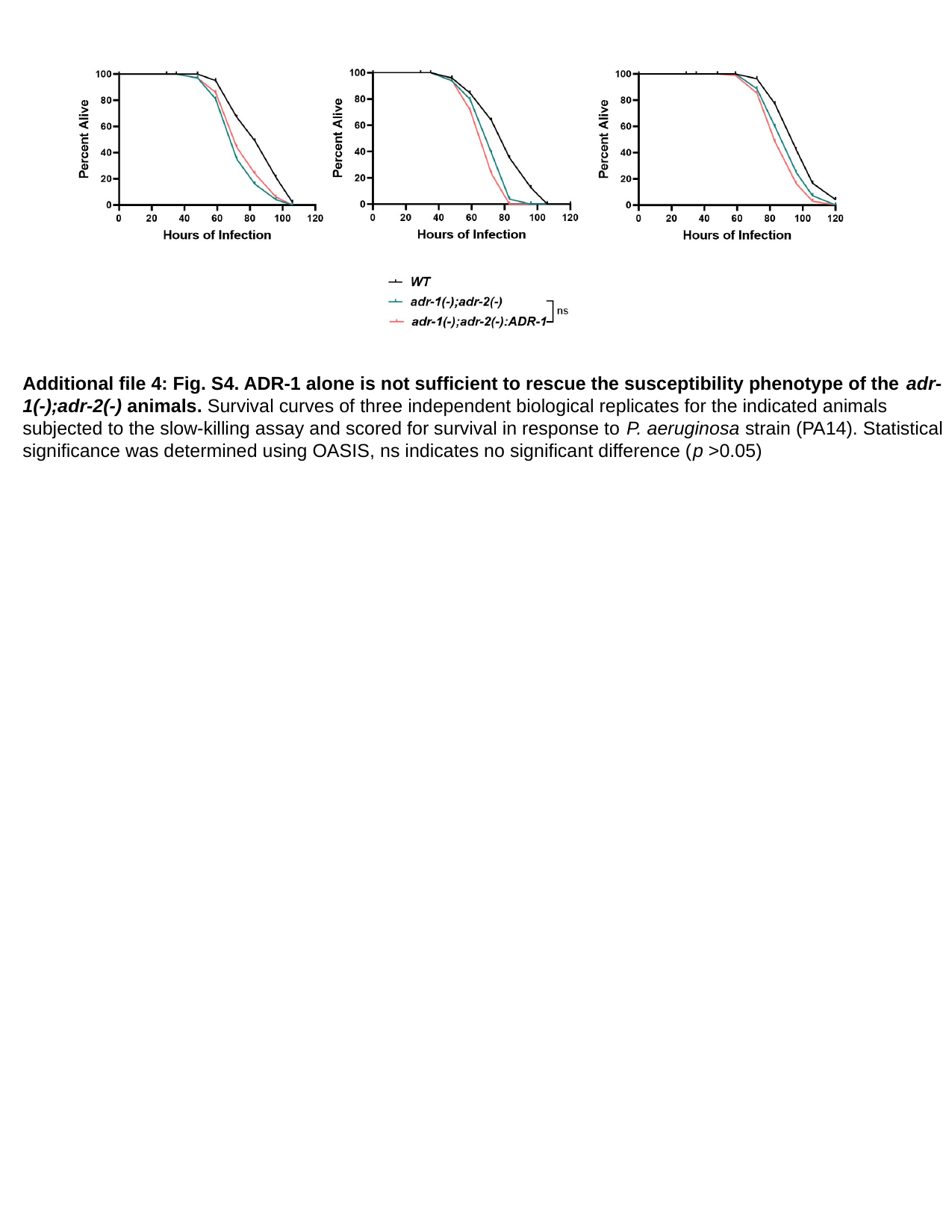

Additional file 4: Fig. S4. ADR-1 alone is not sufficient to rescue the susceptibility phenotype of the adr-1(-);adr-2(-) animals. Survival curves of three independent biological replicates for the indicated animals subjected to the slow-killing assay and scored for survival in response to P. aeruginosa strain (PA14). Statistical significance was determined using OASIS, ns indicates no significant difference (p >0.05)​
​
