# Supplementary material for: ADARs regulate cuticle collagen expression and promote survival to pathogen infection
Source: BMC Biol. 2024 Feb 16;22:37. doi: 10.1186/s12915-024-01840-1 (PMC10870475; doi:10.1186/s12915-024-01840-1)
Supplement: Supplementary file 6 — Additional file 6: Fig. S6. ADR-1 protein expression increases upon P. aeruginosa (PA14) exposure. (Related Fig. 2). Uncropped western blots of all biological replicates. [file 12915_2024_1840_MOESM6_ESM.pptx]

## Slide 1
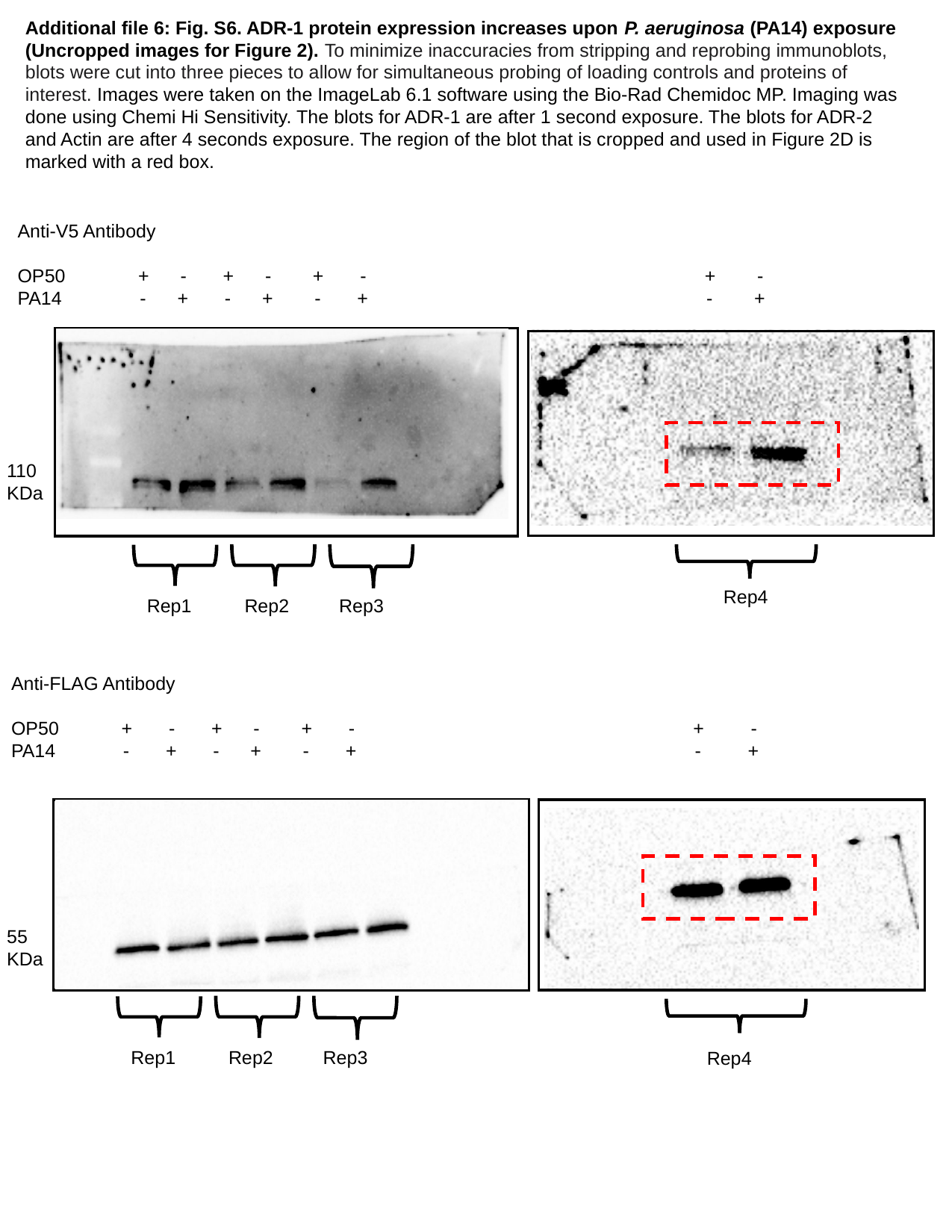

Additional file 6: Fig. S6. ADR-1 protein expression increases upon P. aeruginosa (PA14) exposure (Uncropped images for Figure 2). To minimize inaccuracies from stripping and reprobing immunoblots, blots were cut into three pieces to allow for simultaneous probing of loading controls and proteins of interest. Images were taken on the ImageLab 6.1 software using the Bio-Rad Chemidoc MP. Imaging was done using Chemi Hi Sensitivity. The blots for ADR-1 are after 1 second exposure. The blots for ADR-2 and Actin are after 4 seconds exposure. The region of the blot that is cropped and used in Figure 2D is marked with a red box.
Anti-V5 Antibody
OP50              +      -       +      -        +       -                                                                 +        -
PA14               -      +       -      +        -       +                                                                 -        +
110 KDa
Rep4
Rep1
Rep2
Rep3
Anti-FLAG Antibody
OP50            +       -       +      -        +       -                                                                 +         -
PA14             -       +       -      +        -       +                                                                 -         +
55 KDa
Rep1
Rep2
Rep3
Rep4

## Slide 2
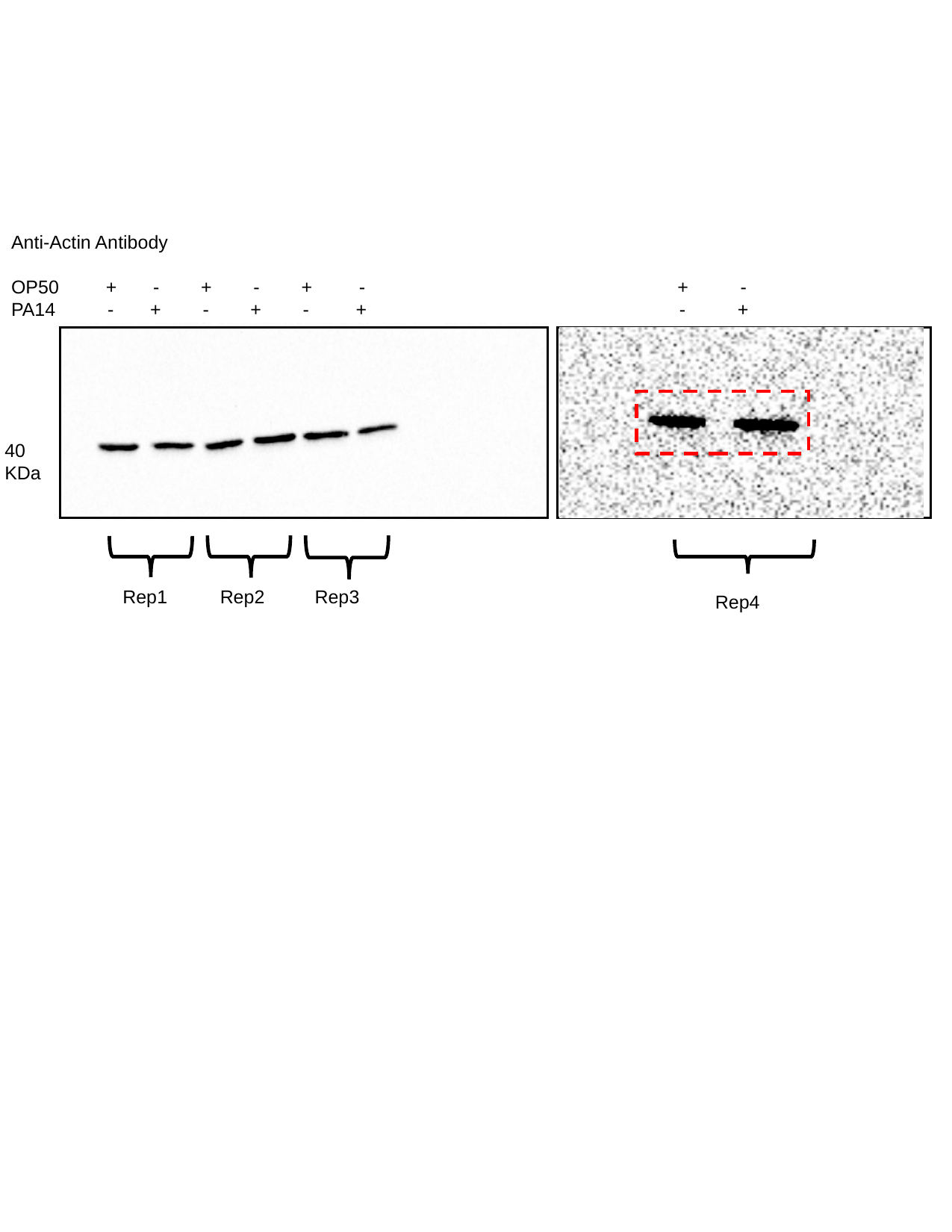

Anti-Actin Antibody
OP50         +       -        +        -        +         -                                                            +          -
PA14          -       +        -        +        -         +                                                            -          +
40 KDa
Rep1
Rep2
Rep3
Rep4
