# Supplementary material for: ADARs regulate cuticle collagen expression and promote survival to pathogen infection
Source: BMC Biol. 2024 Feb 16;22:37. doi: 10.1186/s12915-024-01840-1 (PMC10870475; doi:10.1186/s12915-024-01840-1)
Supplement: Supplementary file 7 — Additional file 7: Fig. S7. ADR-1 RNA binding is required for survival to P. aeruginosa. Survival curves of independent biological replicates for ADR-1 RNA binding mutant animals subjected to the slow-killing assay. [file 12915_2024_1840_MOESM7_ESM.pptx]

## Slide 1
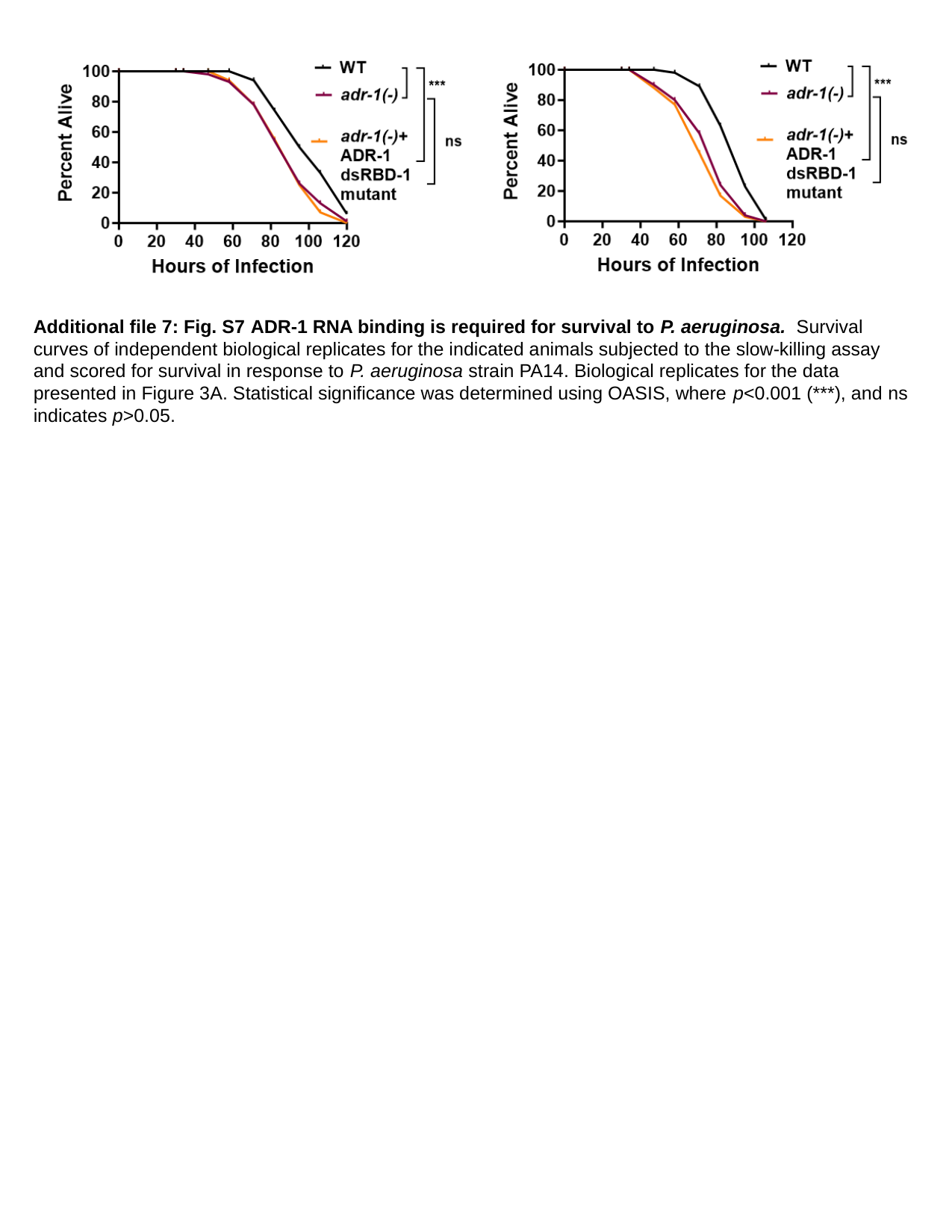

Additional file 7: Fig. S7 ADR-1 RNA binding is required for survival to P. aeruginosa.  Survival curves of independent biological replicates for the indicated animals subjected to the slow-killing assay and scored for survival in response to P. aeruginosa strain PA14. Biological replicates for the data presented in Figure 3A. Statistical significance was determined using OASIS, where p<0.001 (***), and ns indicates p>0.05.
