# Supplementary material for: ADARs regulate cuticle collagen expression and promote survival to pathogen infection
Source: BMC Biol. 2024 Feb 16;22:37. doi: 10.1186/s12915-024-01840-1 (PMC10870475; doi:10.1186/s12915-024-01840-1)
Supplement: Supplementary file 10 — Additional file 10: Fig. S10. Gene set enrichment analysis of 332 misregulated genes in adr-1(-) animals and ADR-1 dsRBD1 mutant animals exposed to PA14. Gene set enrichment analysis using WormCat output and FuncAssociate output. [file 12915_2024_1840_MOESM10_ESM.pptx]

## Slide 1
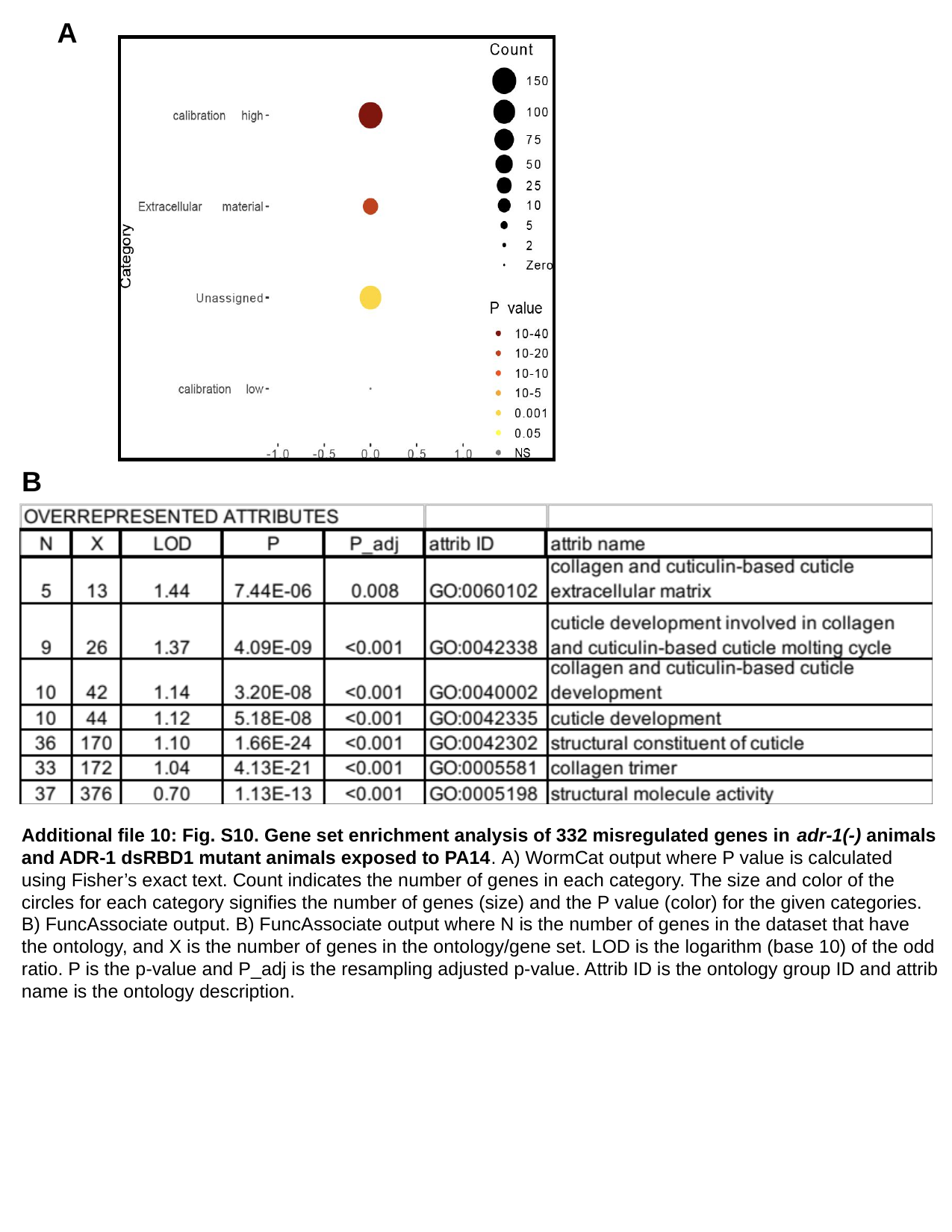

A
B
Additional file 10: Fig. S10. Gene set enrichment analysis of 332 misregulated genes in adr-1(-) animals and ADR-1 dsRBD1 mutant animals exposed to PA14. A) WormCat output where P value is calculated using Fisher’s exact text. Count indicates the number of genes in each category. The size and color of the circles for each category signifies the number of genes (size) and the P value (color) for the given categories. B) FuncAssociate output. B) FuncAssociate output where N is the number of genes in the dataset that have the ontology, and X is the number of genes in the ontology/gene set. LOD is the logarithm (base 10) of the odd ratio. P is the p-value and P_adj is the resampling adjusted p-value. Attrib ID is the ontology group ID and attrib name is the ontology description.
