# Supplementary material for: ADARs regulate cuticle collagen expression and promote survival to pathogen infection
Source: BMC Biol. 2024 Feb 16;22:37. doi: 10.1186/s12915-024-01840-1 (PMC10870475; doi:10.1186/s12915-024-01840-1)
Supplement: Supplementary file 15 — Additional file 15: Fig. S15. Enrichment analysis of common downregulated genes in adr-1(-), adr-2(-) and adr-1(-);adr-2(-) grown on OP50. Enrichment analysis was done using wormcat and FuncAssociate for common downregulated genes. [file 12915_2024_1840_MOESM15_ESM.pptx]

## Slide 1
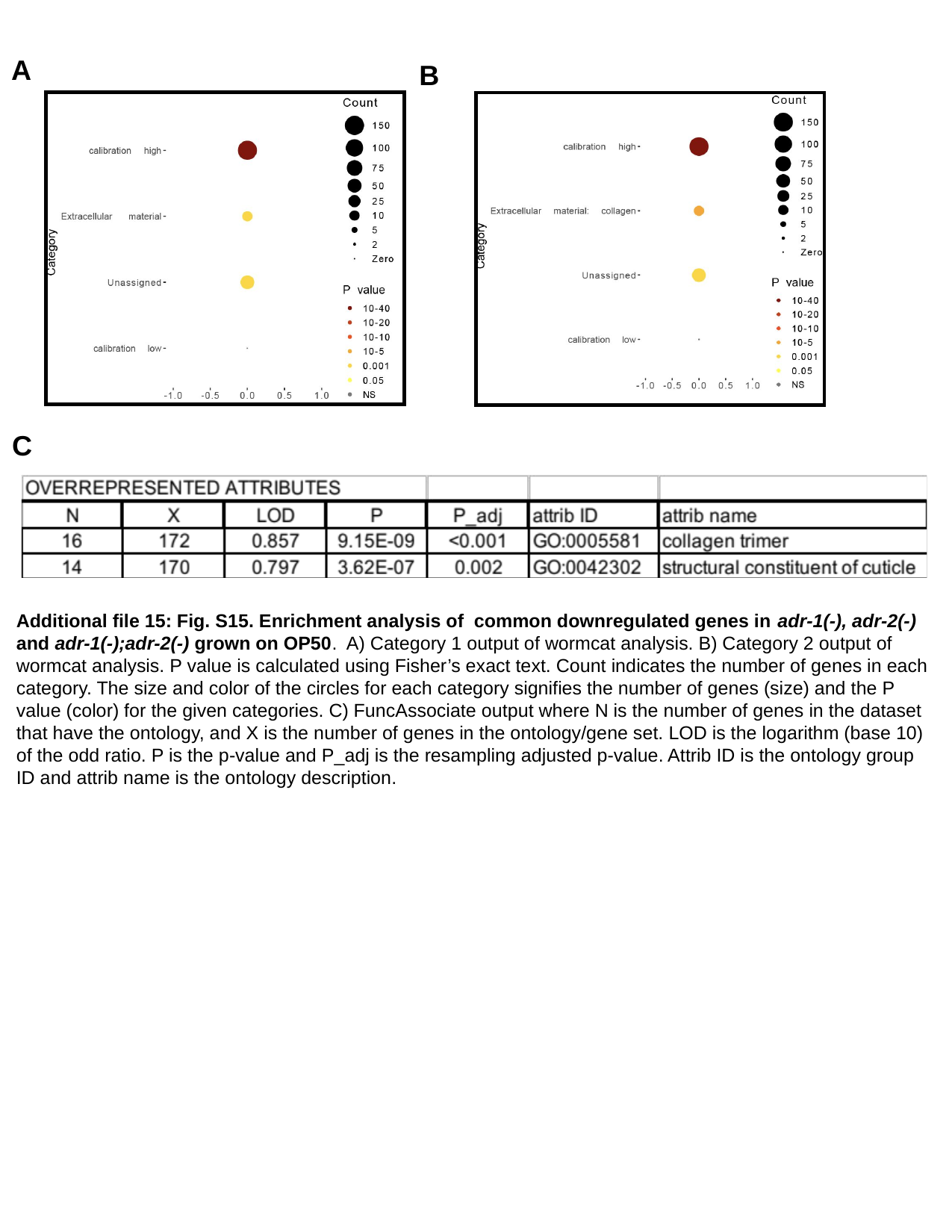

A
B
C
Additional file 15: Fig. S15. Enrichment analysis of  common downregulated genes in adr-1(-), adr-2(-) and adr-1(-);adr-2(-) grown on OP50.  A) Category 1 output of wormcat analysis. B) Category 2 output of wormcat analysis. P value is calculated using Fisher’s exact text. Count indicates the number of genes in each category. The size and color of the circles for each category signifies the number of genes (size) and the P value (color) for the given categories. C) FuncAssociate output where N is the number of genes in the dataset that have the ontology, and X is the number of genes in the ontology/gene set. LOD is the logarithm (base 10) of the odd ratio. P is the p-value and P_adj is the resampling adjusted p-value. Attrib ID is the ontology group ID and attrib name is the ontology description.
