# Supplementary material for: ADARs regulate cuticle collagen expression and promote survival to pathogen infection
Source: BMC Biol. 2024 Feb 16;22:37. doi: 10.1186/s12915-024-01840-1 (PMC10870475; doi:10.1186/s12915-024-01840-1)
Supplement: Supplementary file 16 — Additional file 16: Fig. S16. qRT-PCR validation of downregulated collagen genes from RNA-seq analysis. qRT-PCR quantification of the level of the collagen genes in adr mutant animals. [file 12915_2024_1840_MOESM16_ESM.pptx]

## Slide 1
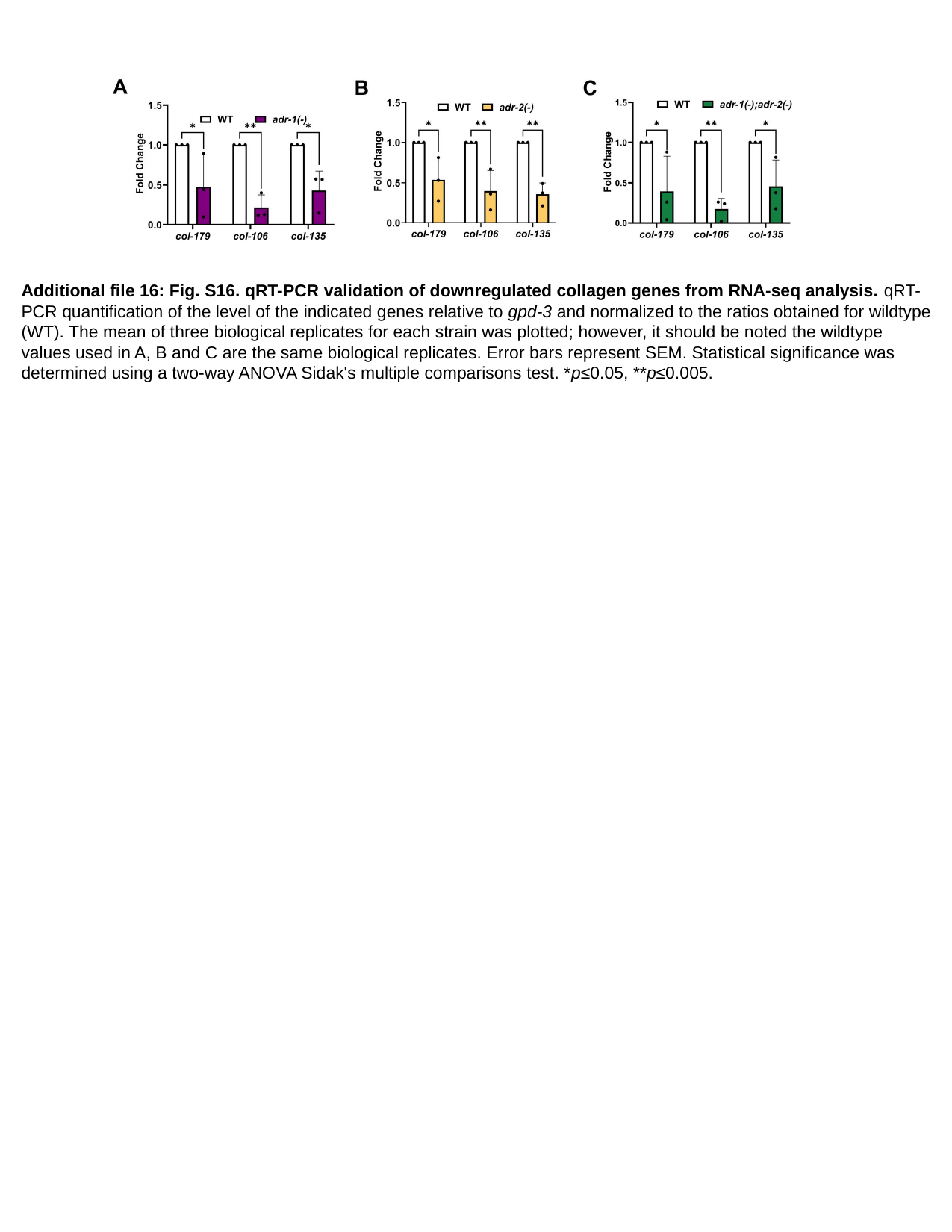

Additional file 16: Fig. S16. qRT-PCR validation of downregulated collagen genes from RNA-seq analysis. qRT-PCR quantification of the level of the indicated genes relative to gpd-3 and normalized to the ratios obtained for wildtype (WT). The mean of three biological replicates for each strain was plotted; however, it should be noted the wildtype values used in A, B and C are the same biological replicates. Error bars represent SEM. Statistical significance was determined using a two-way ANOVA Sidak's multiple comparisons test. *p≤0.05, **p≤0.005.
