# Supplementary material for: ADARs regulate cuticle collagen expression and promote survival to pathogen infection
Source: BMC Biol. 2024 Feb 16;22:37. doi: 10.1186/s12915-024-01840-1 (PMC10870475; doi:10.1186/s12915-024-01840-1)
Supplement: Supplementary file 18 — Additional file 18: Fig. S18. Loss of adrs results in enhanced susceptibility to several pathogenic bacterial species. (Related to Fig. 5). Additional survival curves for the adr-1(-);adr-2(-) animals subjected to the slow-killing assay with S. aureus and S. enterica. [file 12915_2024_1840_MOESM18_ESM.pptx]

## Slide 1
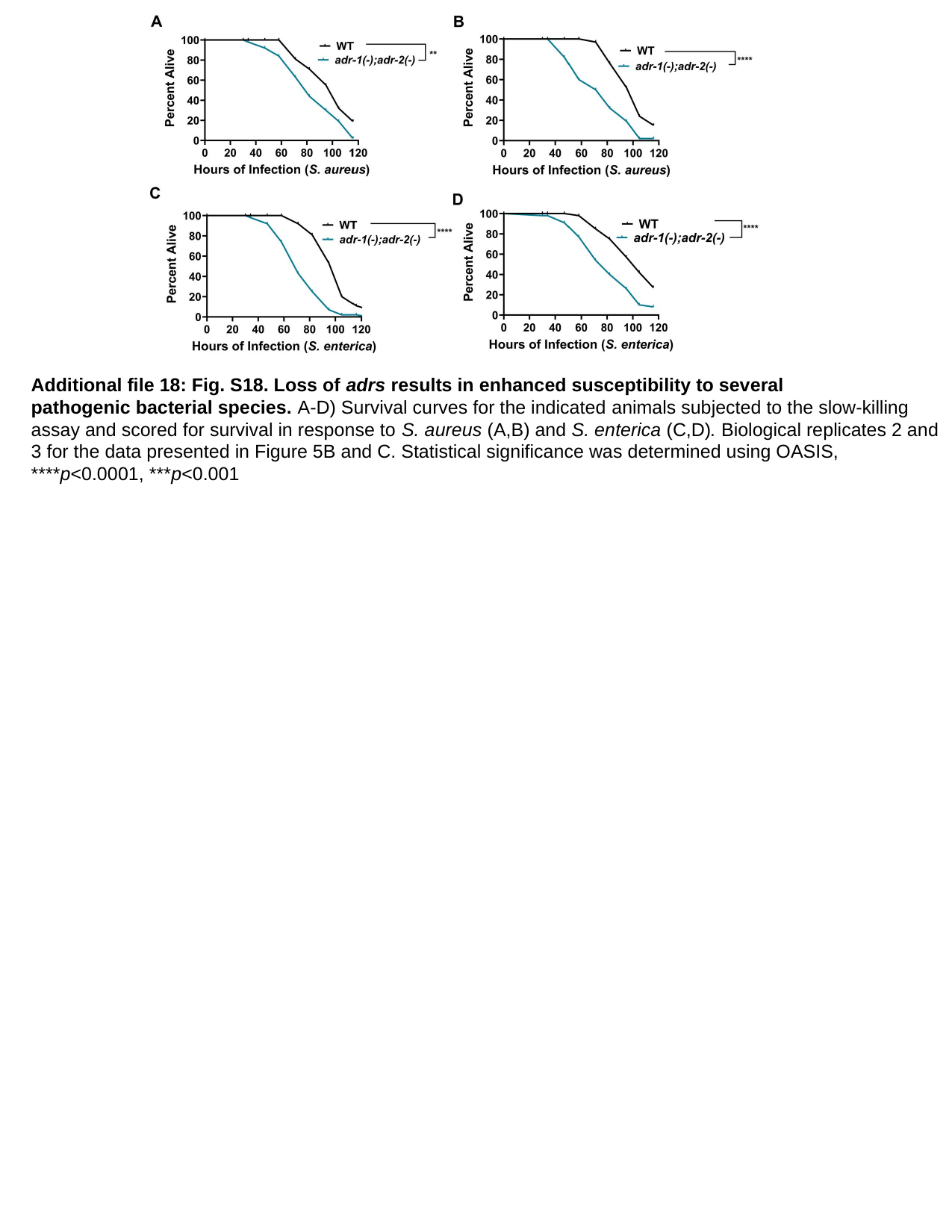

Additional file 18: Fig. S18. Loss of adrs results in enhanced susceptibility to several pathogenic bacterial species. A-D) Survival curves for the indicated animals subjected to the slow-killing assay and scored for survival in response to S. aureus (A,B) and S. enterica (C,D). Biological replicates 2 and 3 for the data presented in Figure 5B and C. Statistical significance was determined using OASIS, ****p<0.0001, ***p<0.001
