# Supplementary material for: ADARs regulate cuticle collagen expression and promote survival to pathogen infection
Source: BMC Biol. 2024 Feb 16;22:37. doi: 10.1186/s12915-024-01840-1 (PMC10870475; doi:10.1186/s12915-024-01840-1)
Supplement: Supplementary file 19 — Additional file 19: Fig. S19. P. aeruginosa bacterial load is comparable in wildtype and adr mutant animals. PA14 bacterial load quantified for wildtype and adr mutant animals. [file 12915_2024_1840_MOESM19_ESM.pptx]

## Slide 1
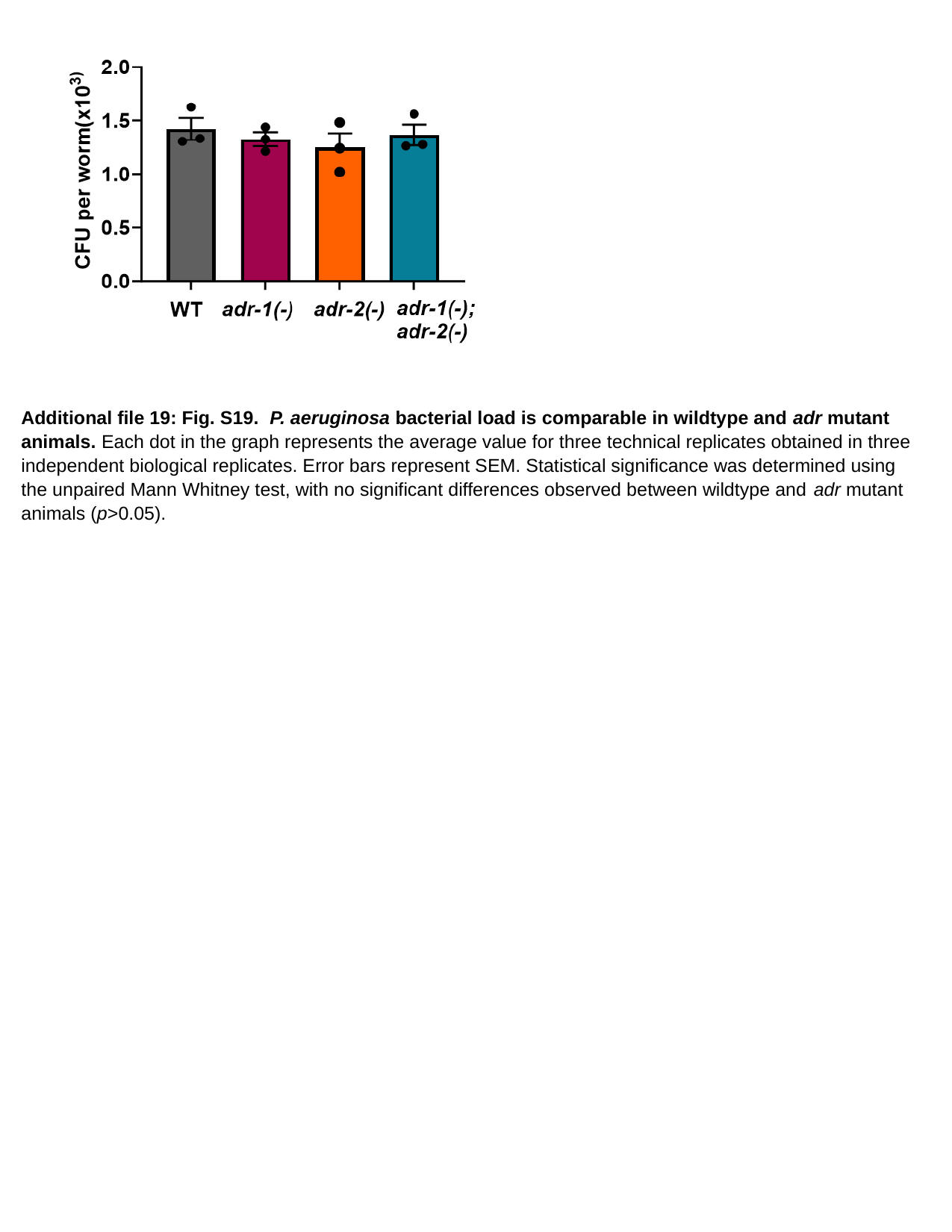

Additional file 19: Fig. S19.  P. aeruginosa bacterial load is comparable in wildtype and adr mutant animals. Each dot in the graph represents the average value for three technical replicates obtained in three independent biological replicates. Error bars represent SEM. Statistical significance was determined using the unpaired Mann Whitney test, with no significant differences observed between wildtype and adr mutant animals (p>0.05).
